# Exploring Disulfide Bridge as a Tool to Improve Pioglitazone’s Neuroprotective Potential: Toward the Development of Prolonged‐Acting MAO‐B/PPARγ Modulators

**DOI:** 10.1002/cmdc.70405

**Published:** 2026-07-29

**Authors:** Filippo Basagni, Maria Luisa Di Paolo, Antonio Laghezza, Giorgio Cozza, Francesco Piazzola, Laura Facci, Emma Marcolin, Elena Roggiolani, Luca Piemontese, Anna Minarini, Morena Zusso, Lisa Dalla Via, Michela Rosini

**Affiliations:** ^1^ Department of Pharmacy and Biotechnology Alma Mater Studiorum‐University of Bologna Bologna Italy; ^2^ Department of Molecular Medicine University of Padova Padova Italy; ^3^ Department of Pharmacy‐Drug Sciences University of Bari Aldo Moro Bari Italy; ^4^ Department of Pharmaceutical and Pharmacological Sciences University of Padova Padova Italy; ^5^ IRCCS San Camillo Hospital Venezia Italy

**Keywords:** disulfide bridge, monoamine oxidase B, neurodegenerative diseases, peroxisome proliferator‐activated receptor γ, pioglitazone

## Abstract

Neurodegenerative diseases’ treatments still represent one of the major unmet medical needs, and their related clinical trials are characterized by one of the highest failure rates. Despite the promising preclinical premises, pioglitazone lies among those drugs that failed clinical translation. It is an antidiabetic drug acting as a PPARγ agonist and later repurposed as a competitive and selective MAO‐B inhibitor featuring polyhedral neuroprotective properties. In pursuing our efforts to broaden the therapeutic potential of pioglitazone toward the neurodegenerative context, we herein developed and preliminarily characterized a new series of glitazone derivatives. Particularly, by means of different connectors, a disulfide covalent warhead was attached to the 5‐benzylthiazolidine‐2,4‐dione head to potentially engage cysteines located in MAO‐B and PPARγ binding sites and turn pioglitazone into a prolonged‐acting modulator. Interestingly, compound **5** emerged as a selective competitive MAO‐B inhibitor and a PPARγ agonist with a very slow dissociation rate, revealing long‐lasting target engagement, albeit exhibiting lower potencies with respect to pioglitazone. This peculiar biological profile resulted in promising antioxidant and anti‐inflammatory properties, laying the ground for future development of covalent pioglitazone derivatives.

## Graphical abstract

Pioglitazone is an antidiabetic drug acting as a peroxisome proliferator‐activated receptor γ (PPARγ) agonist and later repositioned as a monoamine oxidase B (MAO‐B) inhibitor. Despite promising preclinical premises, it showed a lack of efficacy in clinical trials as a treatment for neurodegenerative diseases. Looking to improve its neuroprotective efficacy, we herein report new pioglitazone derivatives as potential prolonged‐acting modulators of the two targets.

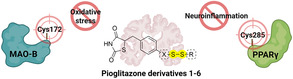

## 1 Introduction

Drug repurposing represents a powerful opportunity for drug development in the field of neurodegeneration, as the extensive pharmacokinetic characterization already available for approved drugs offers the chance to consciously face and bypass brain bioavailability issues, which are a major vulnerability in discovery campaigns toward central nervous system (CNS) therapeutics [1]. Pioglitazone is a test case in this respect. It is a peroxisome proliferator‐activated receptor γ (PPARγ) agonist marketed for the treatment of type 2 diabetes since 1999 and later repositioned as a potent selective and competitive monoamine oxidase B (MAO‐B) inhibitor [2]. These findings immediately suggested its potential use for counteracting neurodegenerative processes, given by merging the anti‐inflammatory effect of a PPAR agonist with the behavior of a MAO‐B inhibitor [3, 4].

MAO‐B is a mitochondrial membrane‐bound enzyme that catalyses the oxidative deamination of biogenic and dietary amines, and its main physiological role is the regulation of the homeostasis of monoamine neurotransmitters. The increased expression and activity of MAO‐B found in the brain affected by some neurodegenerative diseases makes it an attractive drug target. Indeed, in addition to unbalancing the amine neurotransmitters catabolism, the increased level of MAO‐B reaction products (aldehydes and hydrogen peroxide) contributes to the development of oxidative stress, linked to microglial activation and neuroinflammation, which are associated with the neurodegenerative processes [5, 7]. PPARγ is a ligand‐activated transcription factor belonging to the nuclear receptor superfamily, which plays key roles in regulating genes responsible for several physiological processes, such as glucose homeostasis, lipid metabolism, and fat cell differentiation [8]. Furthermore, PPARγ activation has been related to antioxidant, anti‐inflammatory and neuroprotective effects. Particularly, PPARγ mitigates neuroinflammation through regulating Nrf2 and NF‐кB pathways, while its capability to modulate β‐amyloid and tau neurotoxic aggregates’ formation offers prospects for a potential role in tackling neurodegeneration [3, 9].

Based on the blood‐brain barrier (BBB) permeability properties and the overall neuroprotective profile resulting from its biological characterization, pioglitazone was widely studied in several in vitro and in vivo models of neurodegeneration [10, 13]. Particularly, in Alzheimer’s disease (AD) animal models pioglitazone proved to ameliorate synaptic dysfunction and reduce β‐amyloid burden with resulting important cognitive and memory rescue [14, 17]. Unfortunately, all the promising preclinical premises were not confirmed in the following clinical trials. Among the plethora of clinical studies concerning pioglitazone in this respect, the most advanced ones for AD (Phase 3, NCT01931566) and Parkinson’s disease (PD) treatment (Phase 2, NCT01280123) demonstrated lack of efficacy [18, 19].

**FIGURE 1:**
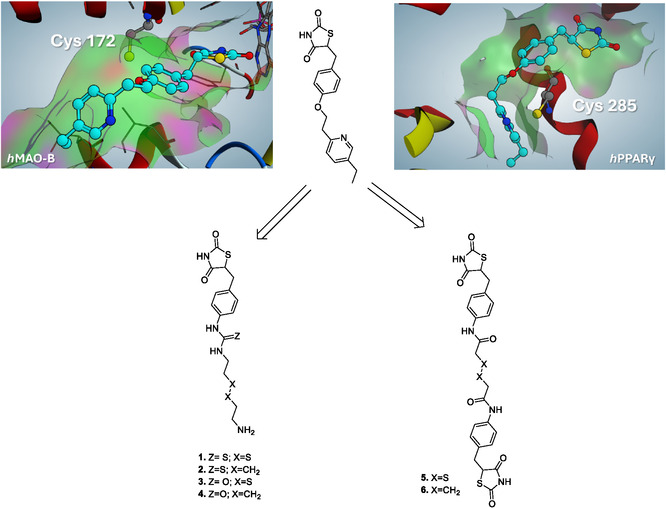
Drug design for pioglitazone derivatives **1–6** bearing a disulfide moiety as potential covalent warhead. The insets illustrate the binding poses of the parent drug, pioglitazone, within the active sites of its two biological targets to highlight the key cysteine residues exploited for the covalent design: the left inset shows pioglitazone within the MAO‐B catalytic pocket, in close proximity to Cys172, while the right inset depicts pioglitazone within the PPARγ ligand‐binding domain, facing Cys285.

**FIGURE 2:**
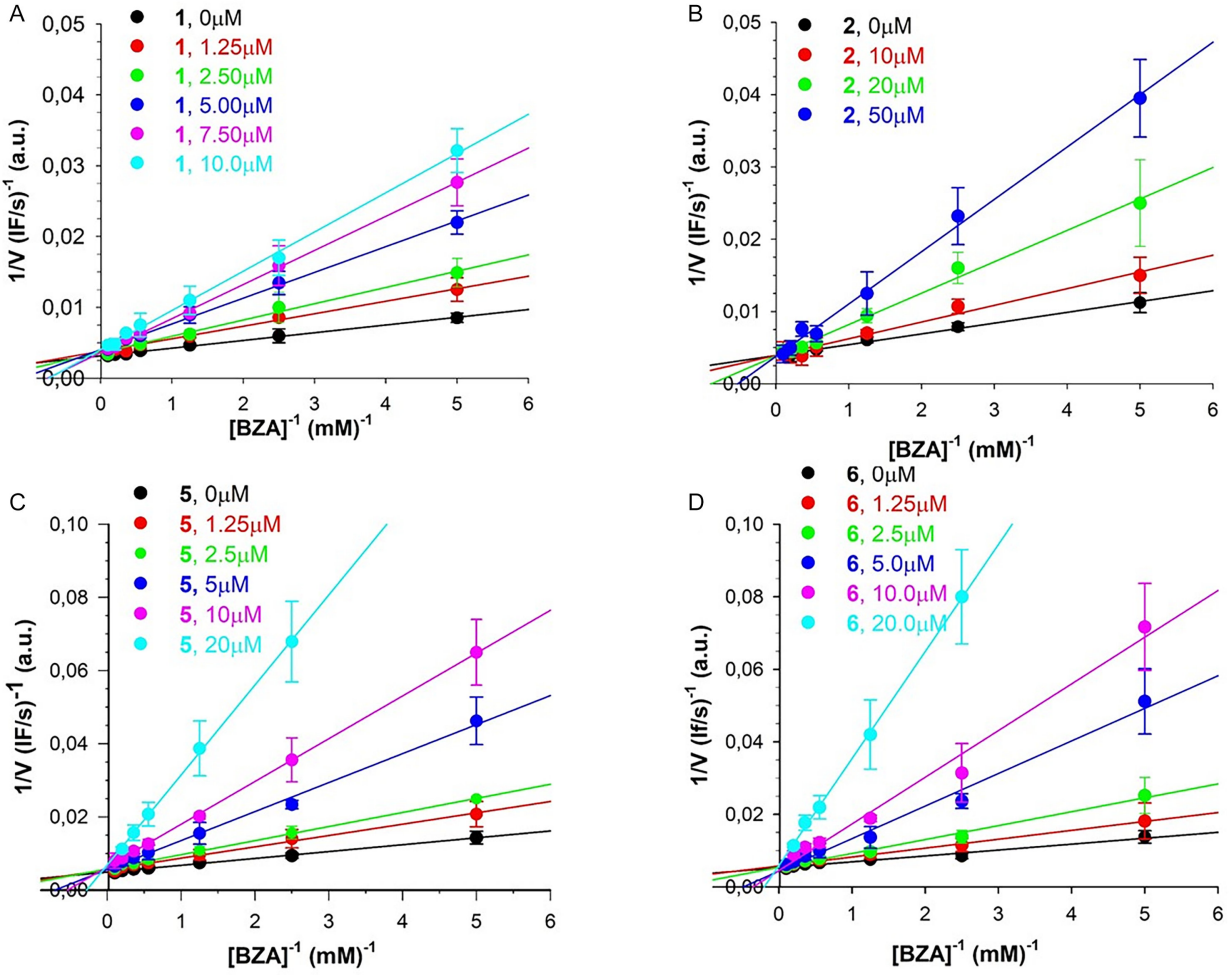
Mechanism of inhibition of **1** versus **2** and **5** versus **6** for *h*MAO‐B. Lineweaver–Burk plots of MAO‐B activity in the absence (•) and in the presence of different concentrations of **1** (A), **2** (B), **5** (C), and **6** (D) compounds. Continuous lines are the result of linear regression analysis of plotted data (*r* ^2^ > 0.98). Data points indicate the mean values ± SD (standard deviation) from at least three independent experiments. The relative inhibition constant values (*K*_i_) were calculated by global fit analysis (GraphPad Prism 9.0 software) and are reported in Table 1.

Thus, given the promising preclinical premises, we aimed to broaden the pharmacological profile of pioglitazone through tuned chemical modification with the ultimate goal of boosting its neuroprotective potential. In this context, we previously developed pioglitazone’s derivatives with an additive antioxidant profile due to the attached (pro)electrophilic moieties, resulting in additional Nrf2 activation and ROS scavenging properties [20]. Herein, seeking the same original purpose, we report the development of a new series of pioglitazone derivatives decorated with a cysteine trapping warhead. Particularly, keeping the 5‐benzylthiazolidine‐2,4‐dione head as crucial for both MAO‐B and PPARγ activities, we inserted in the *para* position of the benzyl moiety different appendages bearing a disulfide bridge with the dual aim of achieving the covalent modulation of two targets and a polyhedral antioxidant profile. Concerning the first point, both MAO‐B and PPARγ hold cysteines proximal to pioglitazone binding pose (i.e., Cys172 and Cys285, respectively) within the two active sites (Figure 1), [2, 21] that can be covalently trapped by the disulfide warhead through thiol‐disulfide exchange reaction; thus, turning selective competitive modulators into targeted covalent ones should enhance their therapeutic potential thanks to the prolonged modulation of the same targets [22]. Furthermore, covalently facing Cys172 in MAO‐B can offer a promising therapeutic alternative which could overcome liabilities related to common FAD‐directed irreversible inhibitors [23], while ensuring selectivity over MAO‐A due to its nonconserved nature. Concurrently, Cys285 of PPARγ is particularly sought after in recent years, albeit with low consideration for the development of agonists as a potential intervention in neurodegenerative diseases [24]. In this context, we previously achieved MAOs’ inactivation by means of disulfide‐bearing polyamines with different selectivity profiles depending on their inner backbone [25]. Additionally, the disulfide‐bearing tethers is supposed to confer additional neuroprotective properties thanks to induced rising free thiols concentration at cellular level, that can exert a direct antioxidant effect [26, 27]. Based on these premises, we developed two classes of pioglitazone derivatives enriched with disulfide‐containing appendages: compounds **1** and **3**, where a cystamine tail is conjugated to the pioglitazone head by means of a thiourea or urea group, respectively, and compound **5**, where the inner dithiodiglycolic tether is interposed between the same two side glitazone functions (Figure 1). Following a simplified covalent tethering approach [28], different linking moieties (i.e., (thio)urea and amide) and symmetry (i.e., mono‐ and di‐derivatization of 5‐benzylthiazolidine‐2,4‐dione head) were explored to identify the optimal position for the covalent interaction between the reactive disulfide bridge and cysteines within the binding sites. Furthermore, to assess the biological role of the inserted disulfide moiety within this class of compounds, the respective methylene‐based counterparts were developed (i.e., compounds **2**, **4** and **6**, Figure 1) and tested in parallel. After preliminary screening of all new compounds on MAO‐B and PPARγ, we assessed their putative covalent‐based mechanism of action toward the targets of interest. Finally, the most promising compounds of the series were evaluated for their antioxidant and anti‐inflammatory profile in different cellular models.

## 2 Results and Discussion

### 2.1 Chemistry

**SCHEME 1:**
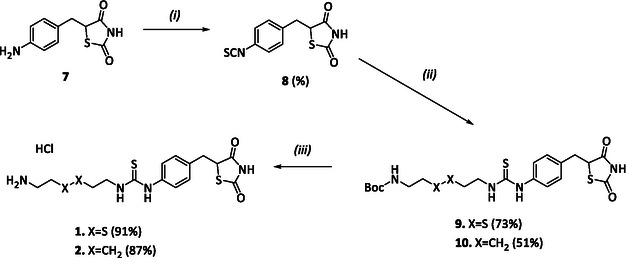
Reagents and conditions: *(* *i* *)* TCDI, DMF, rt, 2 h; *(* *ii* *)* N‐Boc‐diamine, Et_3_N, DMF, rt, 2 h; *(* *iii* *)* HCl 4 M in dioxane, −20 °C, 2 h.

**SCHEME 2:**
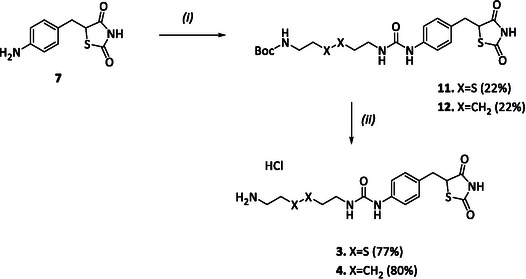
Reagents and conditions: (*i*) a. Et_3_N, THF, PhOCOCl, rt, 10 min; b. *N*‐Boc‐diamine, Et_3_N, THF, rt, 24 h; (*ii*) HCl 4 M in dioxane, −20 °C, 2 h.

**SCHEME 3:**
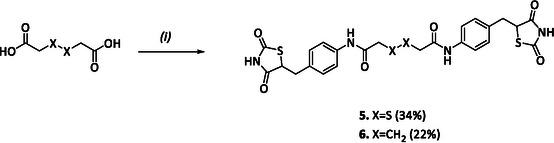
Reagents and conditions: (*i*) a. HOBt, EDC, DCM, 0 °C, 30 min; b. **7**, DMF, rt, 24 h.

Compound **7**, *N*‐Boc‐cystamine and *N*‐Boc‐1,6‐hexanediamine were synthesized as previously reported [12, 29, 30]. The synthetic strategies exploited to achieve final compounds **1–6** are reported in Schemes 1–3 and all depart from aniline intermediate **7**. To obtain thioureas **9** and **10**, the aniline function of **7** was previously converted into isothiocyanate in compound **8** and then underwent nucleophilic addition from respective *N*‐Boc‐diamines (Scheme 1), while phenyl carbamate in situ‐formed intermediates were employed to get to ureas **11** and **12** (Scheme 2). In both cases, final acidic Boc deprotection occurred to obtain final compounds **1–4** as hydrochloride salts. Differently, compounds **5** and **6** were prepared by means of a double amide coupling reaction between appropriate dicarboxylic acids (i.e., adipic and dithiodiglycolic acid) and two **7**’s moieties (Scheme 3).

### 2.2 Activities and Kinetic Characterization of New Derivatives Against MAO‐B

At first, compounds **1–6** were tested as potential MAO substrates, and activity was compared to standard substrates (i.e., *p*‐tyramine (Tyr) for *h*MAO‐A and benzylamine (BZA) for *h*MAO‐B) at the same concentration (200 µM), but all compounds resulted almost inactive in this respect (data not shown). Therefore, we further evaluated the inhibitory capabilities of the compounds toward MAOs. First, the reversibility of compounds–MAOs interaction and consequent inhibition were assessed through a jump dilution assay [31], to evaluate the potential covalent interaction between disulfide moiety and MAOs' active site (Table 1). Particularly, after preincubation of MAOs with 200 µM compound for 20 min, the solution was diluted 1/100 (2 µM final concentration), substrate was added, and the MAO activity was measured (Figure S1). These recovered enzyme activities were compared to those of nonpreincubated samples and in the presence of the same compound concentration (2 µM). Clorgyline and selegiline were used as reference irreversible inhibitors of MAO‐A and MAO‐B, respectively. Harmine and safinamide were included as standard selective and competitive inhibitors of MAO‐A and MAO‐B, respectively. As reported in Table 1, all compounds bearing a disulfide bridge exerted greater inhibitory effects than their counterparts, with similar magnitudes to those of the nonpreincubated samples and very far from the employed irreversible references, suggesting that new derivatives do not irreversibly inactivate MAOs. This behavior was further confirmed by dialysis experiments of MAO‐B with **5**, the most effective compound of the series on this isoform, compared to selegiline: after dialysis of MAO‐B preincubated with **5**, 98% of its enzymatic activity was recovered (in comparison to the control sample), while only 4.5% of MAO‐B activity was recovered after selegiline treatment. For standard competitive inhibitors, harmine and safinamide, similar magnitudes of activity were found both for the preincubated and diluted samples and the nonincubated one, confirming their known reversibility.

**TABLE 1:** *h*MAO inhibitory activities for compounds 1–6 with clorgyline and selegiline (standard irreversible inhibitors), harmine and safinamide (standard competitive inhibitors), and pioglitazone as reference compounds.

Compound	*h*MAO‐A			*h*MAO‐B		
	Recovered activity after preincubation and dilution, %^a^	Activity at 2 µM (no preincubation), %^b^	*K*_i_, µM	Recovered activity after preincubation and dilution, %^a^	Activity at 2 µM (no preincubation), %^b^	*K*_i_, µM
**1**	47 ± 13	54 ± 9	56 ± 3	63 ± 15	57 ± 4	1.9 ± 0.6
**2**	75 ± 13	79 ± 11	>100	87 ± 7	82 ± 13	12 ± 5
**3**	73 ± 17	88 ± 7	>100	85 ± 3	63 ± 7	14 ± 4
**4**	99 ± 5	89 ± 10	>100	92 ± 8	97 ± 11	23 ± 7
**5**	48 ± 9	61 ± 12	>100	52 ± 4	60 ± 4	1.8 ± 0.3
**6**	81 ± 7	72 ± 14	>100	74 ± 4	55 ± 10	1.6 ± 0.4
Clorgyline	2 ± 1	59 ± 11	n.d.	n.d.	n.d.	n.d.
Selegiline	n.d.	n.d.	n.d.	4 ± 1	7 ± 1	n.d.
Harmine^c^	49 ± 11^c^	65 ± 10^c^	0.008 ± 0.003	n.d.	n.d.	n.d.
Safinamide^c^	n.d.	n.d.	n.d.	85 ± 7^c^	103 ± 11^c^	0.025 ± 0.06
Pioglitazone	n.d.	n.d.	>>100	n.d.	n.d.	0.061 ± 0.018

*Note*: Values are mean ± SD (standard deviation) from at least three independent experiments (each performed in triplicate).

Abbreviation: n.d., not determined.

a The recovery of MAO activity was determined at the end of 20 min of preincubation of enzyme with 200 µM of the tested compound (*T* = 37 °C), after dilution of the sample (1/100, 2 µM final concentration) and addition of substrate.

b In the nonpreincubated samples, the MAO activity was determined directly in the presence of 2 µM compound.

c For standard competitive inhibitors, the preincubation was performed at 500 nM concentration; after dilution and in the nonincubated samples, the final concentration was 5 nM. Substrates were: 1 mM Tyr and 10 mM BZA for MAO‐A and MAO‐B, respectively. The percentage of MAO activity was calculated with respect to the control sample run in parallel but in the absence of any compound. The inhibition constant values (*K*_i_) were calculated by global fit analysis (GraphPad Prism 9.0 software) of the data of reaction rate (*V*) at various substrate concentrations, in the presence of various concentrations of the tested compound.

Therefore, we assessed the inhibition constants and mechanism of action of tested inhibitors exploiting pioglitazone as reference compound (Table 1). All new pioglitazone derivatives behaved as MAO‐B competitive inhibitors, that is, they bind to the active site of the free enzyme. Indeed, the Lineweaver–Burk plot (1/V vs. 1/[S]) reported as examples in Figure 2 clearly shows an increase in the apparent Michaelis–Menten constant values (*K*_M_) (being −1/*K*_M_ the intercept on the *x*‐axis) and no changes in the *V*_max_ values (being 1/*V*_max_ the intercept on the *y*‐axis). Particularly, among thioureas and ureas, disulfide‐bearing derivatives demonstrated a notable improved potency over their methylene‐bridged analogs (*K*_i_ = 1.9 µM of **1** vs. *K*_i_ = 12 µM of **2** and *K*_i_ = 14 µM of **3** vs. *K*_i_ = 23 µM of **4**) as well as for thiourea over urea counterparts (*K*_i_ = 1.9 µM of **1** vs. *K*_i_ = 14 µM of **3** and *K*_i_ = 12 µM of **2** vs. *K*_i_ = 23 µM of **4**). Differently, regarding the symmetric compounds **5** and **6**, the presence of a disulfide moiety did not influence the MAO‐B inhibitory potency, emerging with a low micromolar *K*_i_ for both compounds (*K*_i_ = 1.8 µM of **5** vs. *K*_i_ = 1.6 µM of **6**). Notably, none of the introduced modifications interfered with the MAO‐B selectivity of pioglitazone, given that all compounds resulted as very weak MAO‐A inhibitors (*K*_i_ > 100 µM), except for **1** (*K*_i_ = 56 µM), which, toward this isoform, behaved as an uncompetitive inhibitor (Figure S2).

**FIGURE 3:**
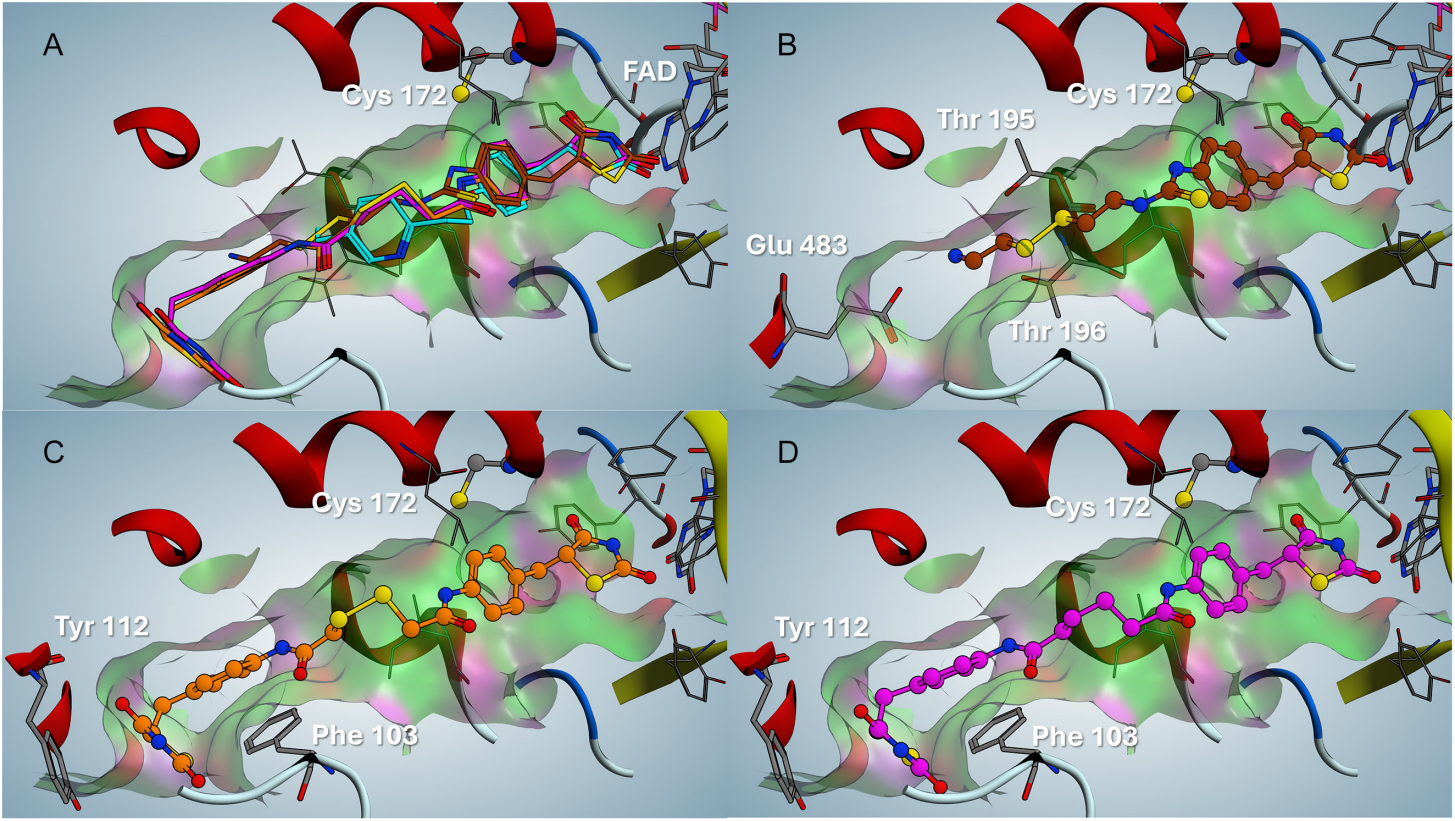
Molecular docking simulations of the synthesized compounds within the *h*MAO‐B catalytic pocket. (A) Superimposition of the predicted binding poses of compound **1** (brown), compound **5** (orange), and compound **6** (purple) with the reference structure of pioglitazone (cyan). The molecules span the length of the cavity, extending from the FAD cofactor toward the entrance of the binding pocket. (B) Detailed binding mode of compound **1**, highlighting the engagement of its terminal amino group with the hydrophilic patch formed by Thr195, Thr196, and Glu483. (C) Binding pose of the disulfide‐bearing compound **5**, emphasizing its extended conformation and the additional π–stacking interactions with Phe103 and Tyr112. (D) Predicted binding pose of the carba‐analog compound **6**, showing a spatial occupation very similar to **5**. In all panels, the interaction surface of the binding pocket is mapped according to its chemical properties: hydrophobic regions are depicted in green, while polar/hydrophilic regions are colored in violet.

Based on these experimental readouts, computational investigations were conducted on the most potent MAO‐B inhibitors of the series (Figure 3). Compounds **1**, **5**, and **6**, inserted into the catalytic pocket of *h*MAO‐B, show significant spatial overlap with the binding pose of pioglitazone, from which they are structurally derived (Figure 3A). Compared to pioglitazone, the described molecules tend to occupy a larger volume of the MAO‐B binding pocket. They extend from the FAD cofactor, where the positioning of the thiazolidinedione head group is conserved, to the entrance of the catalytic pocket. This extended occupation is particularly evident for **5** and **6**, which span over 23 Å, whereas **1** reaches up to 19 Å, compared to the 15 Å spanned by pioglitazone. Although this broader pocket occupancy does not translate into improved inhibitory performance compared to pioglitazone, it demonstrates that specific regions of the catalytic pocket can be efficiently explored from a chemical standpoint. These areas are therefore potentially useful for enhancing both the potency and the selectivity of future candidate molecules.

Indeed, **1**, **5**, and **6** optimally exploit the hydrophobic nature of the MAO‐B catalytic pocket, specifically interacting with residues Ile198, Leu171, Leu167, Ile316, Tyr326, Phe168, Ile199, Leu164, and Trp119. From this position, **1** engages a hydrophilic patch, comprising Thr195, Thr196, and Glu483, for interaction with its terminal amino group (Figure 3B). Conversely, compounds **5** and **6**, which share a highly similar structure, additionally exploit Phe103 and Tyr112 to form π–stacking interactions with the 5‐benzylthiazolidine‐2,4‐dione tail (Figure 3C,D).

To note, based on the pose suggested by molecular docking for this class of compounds, the spatial positioning of Cys172 within the pocket is incompatible with a disulfide exchange reaction, further corroborating the purely competitive enzyme inhibition mechanism as previously discussed.

Finally, the most potent inhibitors of series **1**, **5**, and their analogs without the disulfide moiety were tested to confirm their MAO inhibitory capability in lysates from rat astrocytes, a cellular‐like environment. Even though rat and human MAOs may display slight differences in substrate and inhibitor specificity [32, 33], rat astrocytes have been chosen because they play an important role in neuroinflammatory and neurodegenerative processes [34, 35]. Furthermore, astrocytes express mainly the MAO‐B isoform, whose level was found to increase significantly during neurodegenerative progression [5, 36]. From results reported in Table 2 and shown in Figure S3, compound **5** was found to be the most effective MAO inhibitor of the series, exhibiting a low micromolar IC_50_ in cell lysates (IC_50_ = 0.8 µM). This effect is attributable to the MAO‐B isoform; indeed, the percentage of residual MAO activity at high compound concentrations is likely due to the MAO‐A isoform, which was found to be unaffected by these compounds, consistent with findings on the human MAOs (for details see Figure S3). Conversely, compounds **1**, **2** and **6** showed similar potencies, with IC_50_ values ranging from 6 to 7.8 µM. These data indicate that compound **5** is the most potent MAO‐B inhibitor even against the rat enzyme and remains effective within a complex cellular matrix such as astrocyte lysates.

**TABLE 2:** IC_50_ values of some pioglitazone derivatives and pioglitazone for the inhibitory effect on MAO activity in lysates from rat astrocytes.

Compound	**IC**_**50**_ **, µM**
**1**	7.8 ± 0.8
**2**	6.0 ± 0.7
**5**	0.8 ± 0.1
**6**	6.4 ± 2.3
Pioglitazone	0.014 ± 0.003

*Note*: IC_50_ values are the results of three independent experiments (mean ± SD). Each value of IC_50_ is the result of best fitting of the “inhibitor concentration versus response (three parameters)” equation to the experimental data (percentage of residual MAO activity versus compound concentration), by using the GraphPad Prism 9.0 software.

### 2.3 Investigation of PPARγ Activity Profiles of New Compounds

In parallel, compounds **1**−**6** were evaluated in vitro for their agonist activity toward the human PPARγ (*h*PPARγ) subtype by employing the GAL4‐PPAR transactivation assay. For this purpose, GAL4‐PPAR chimeric receptors were expressed in transiently transfected HepG2 cells according to a previously reported procedure [37]. In particular, the results obtained were compared with corresponding data for pioglitazone used as reference compound. Maximum fold induction obtained with the reference agonist (pioglitazone at 2 µM) was defined as 100%. Only for compounds showing efficacy higher than 10%, the EC_50_ values were determined. As reported in Table 3 and Figure S4, thiourea and urea derivatives generally demonstrated very low activities in this respect, and only disulfide‐bearing compounds **1** and **3** indicated a weak agonist effect. Notably, disulfide‐bearing compound **5** emerged as the only analog of the series with a full agonist profile, albeit with lower potency in respect to glitazone references, with the corresponding carba‐analog **6** behaving as a weak agonist in a similar manner to **1** and **3**. Then, we evaluated the most active compound **5** and its counterpart **6** to see if the introduced modifications on the pioglitazone scaffold affected their selectivity over PPARα and PPARδ isoforms (Figure S5). At the tested concentrations (i.e., 25 and 100 µM), compounds **5** and **6** confirmed their preferential activity on PPARγ, with a very moderate effect with respect to the other isoforms (Emax < 20% at 100 µM).

**TABLE 3:** Biological activity of compounds 1–6 on PPARγ with pioglitazone as reference compound.

Compound	PPARγ	
	**Emax ± SEM, %** a	**EC**_**50 **_ **± SEM, µM**
**1**	13 ± 1	11 ± 2
**2**	i	n.d.
**3**	14 ± 2	26 ± 4
**4**	i	n.d.
**5**	75 ± 5	14.6 ± 2.4
**6**	25 ± 2	26 ± 1
Pioglitazone	100 ± 8	0.365 ± 0.185

*Note*: All data are the results of three separate experiments performed in triplicate.

Abbreviations: SEM, standard error of the mean; n.d., not determined.

a Efficacy values were calculated as a percentage of the maximum obtained fold induction with the reference compounds; i = inactive at concentration up to 100 μM.

**FIGURE 4:**
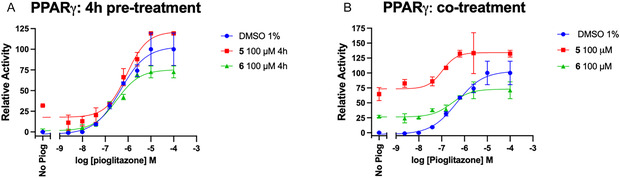
Mechanism of action study of compounds **5** and **6** on PPARγ. Pioglitazone dose‐response in transfected HepG2 cells: (A) cells were preincubated for 4 h with medium containing compounds **5** and **6** (100 µM) or 1% DMSO, followed by a serum‐free medium wash prior to pioglitazone treatment; (B) cells were co‐treated with pioglitazone and a fixed concentration of compounds **5** and **6** (100 µM) or 1% DMSO, without washout.

Due to the important differences that arose between compounds **5** and **6** regarding activity on PPARγ, we further investigated their mechanism of action to evaluate the potential formation of a covalent adduct. Particularly, we determined PPARγ activity at increasing pioglitazone concentrations in experimental conditions of 4 h pretreatment and washout or cotreatment with compounds **5** and **6** at 100 µM when compared with the untreated control (Figure 4). In pretreatment assay, after 4 h incubation with selected compounds, culture medium was removed and substituted with one containing increasing pioglitazone concentrations, leaving other 20 h under incubation before activity measurement. In this case, compound **5** maintained a prolonged basal activity higher than compound **6** and control, even 20 h after medium change, compatible with a putative underlying covalent interaction with the target (Figure 4A). The same trend was maintained upon pioglitazone addition, resulting in an additive effect, given that a portion of receptors was already stably activated by **5**. Notably, this effect was even amplified in cotreatment conditions (Figure 4B). The persistence of compound **5** activity after washout suggests either a very slow dissociation rate or a (pseudo‐)irreversible interaction with PPARγ. However, further studies will help to clarify the molecular basis of this prolonged interaction. Conversely, pretreatment with compound **6** returned the activity to basal level after washout, indicating a reversible binding mode, while in cotreatment it acted as a partial agonist and in the presence of high pioglitazone concentrations led to reduced activity as they compete for the same binding site (competitive antagonism).

**FIGURE 5:**
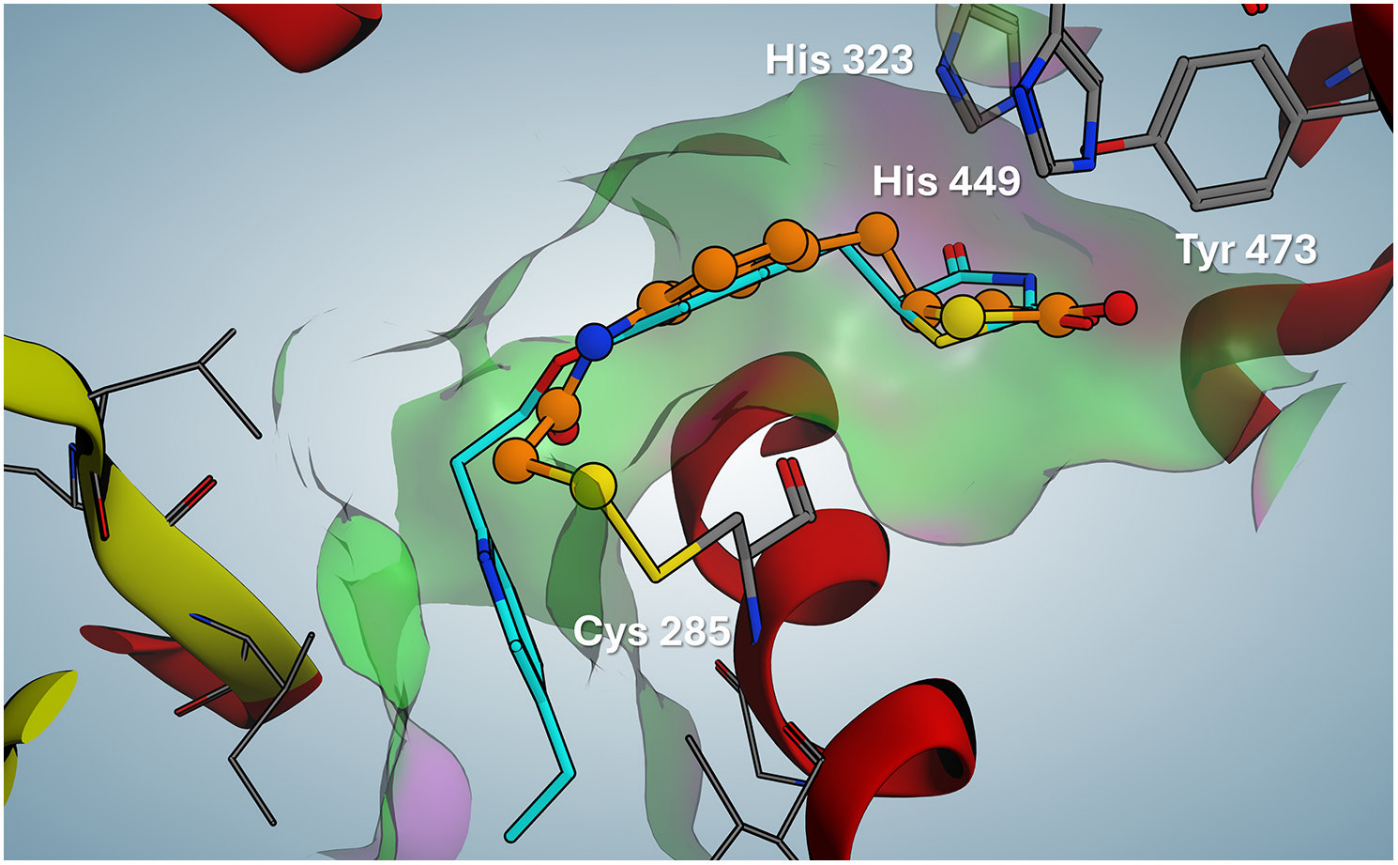
Covalent docking simulation of compound **5** within the *h*PPARγ ligand‐binding domain**.** The predicted binding pose of the “cleaved” form of compound **5** (orange) is shown superimposed with the reference agonist pioglitazone (cyan). The model illustrates the significant spatial overlap of the thiazolidinedione head with pioglitazone, engaging key polar residues such as His323, His449, and Tyr473. Furthermore, the distance and geometry of the inner sulfur of compound **5** are compatible with the formation of a covalent disulfide bond with the reactive Cys285 residue. The interaction surface of the binding pocket is colored to highlight hydrophobic regions in green and polar/hydrophilic regions in violet.

In this case, docking simulations revealed that the distance of compound **5**’s inner sulfur is compatible with the formation of a disulfide bond with Cys285 of PPARγ, albeit with partial conformational strain at the amide group level (Figure 5). Here again, the structural overlap with pioglitazone is significant, involving multiple interactions between His449, His323, Tyr473, and the thiazolidine‐2,4‐dione head, whereas residues Ile326, Leu469, Phe282, Phe363, and Met364 delineate the hydrophobic region that accommodates the remainder of the molecular scaffold. Additionally, the covalently bound halved molecule of **5** accommodated better within the binding pocket in respect to the intact one, due to steric hindrance reasons. This finding is entirely consistent with the agonistic behavior of **5** that emerged from mechanistic investigations in PPARγ transactivation assays and is further confirmed by the low activity of **6** in this respect. Further insights aiming to define the covalent engagement mechanism at the molecular level are ongoing and will be reported in due course.

### 2.4 Cell Viability

Before going into deeper biological investigations at the cellular level, we tested the effect on cell viability of the most potent compounds of the series (i.e., **1**, **5** and their homologs lacking the disulfide moiety, **2** and **6**) on the human tumorigenic LN229 cell line (a glioblastoma cell model) and on C20 (an immortalized human microglial cell line). All tested compounds did not affect the cell viability at both 10 and 20 µM concentrations (Table S1 for results with 20 µM concentration). Furthermore, at all tested concentrations (1–100 µM) used for PPARγ assay in the HepG2 cell line demonstrated no relevant cytotoxicity (data not shown), confirming an overall safety profile.

### 2.5 Antioxidant and Anti‐Inflammatory Properties of Most Promising Compounds

Oxidative stress and inflammation represent two key intertwined pathological events responsible for the onset and progression of neurodegenerative processes [38]. In this context, the abnormal MAO‐B activity leads to increased ROS production and astrogliosis; thus, its inhibition directly mitigates oxidative stress conditions, while PPARγ modulates the transcription of cytoprotective genes, offering prospects for multimodal anti‐inflammatory and antioxidant effects [6, 8, 39]. Furthermore, even if disulfide bonds are generally considered as products of oxidation rather than active antioxidants, recent studies highlights that a disulfide moiety can function *per se* as an active antioxidant by producing highly reactive intermediates [40, 41]. Therefore, by means of an in vitro assay, compounds **1** and **5** and their methylene‐bridged analogs (**2** and **6**) were first evaluated for their potential effect in protecting the oxidation of 2ʹ,7ʹ‐dichlorofluorescein (DCF) induced by ABIP (2,2ʹ‐azobis[2‐(2‐imidazolin‐2‐yl)propane]), a water‐soluble azo compound used as a radical initiator. Results reported in Table S2 clearly support that among the tested new derivatives and pioglitazone itself, only **1** and **5**, containing the disulfide moiety, have the ability to reduce the oxidation rate of DCF (i.e., 47% and 65%, respectively), even if they are less potent than quercetin (i.e., no ROS detected), a standard antioxidant compound exploited for this study.

**FIGURE 6:**
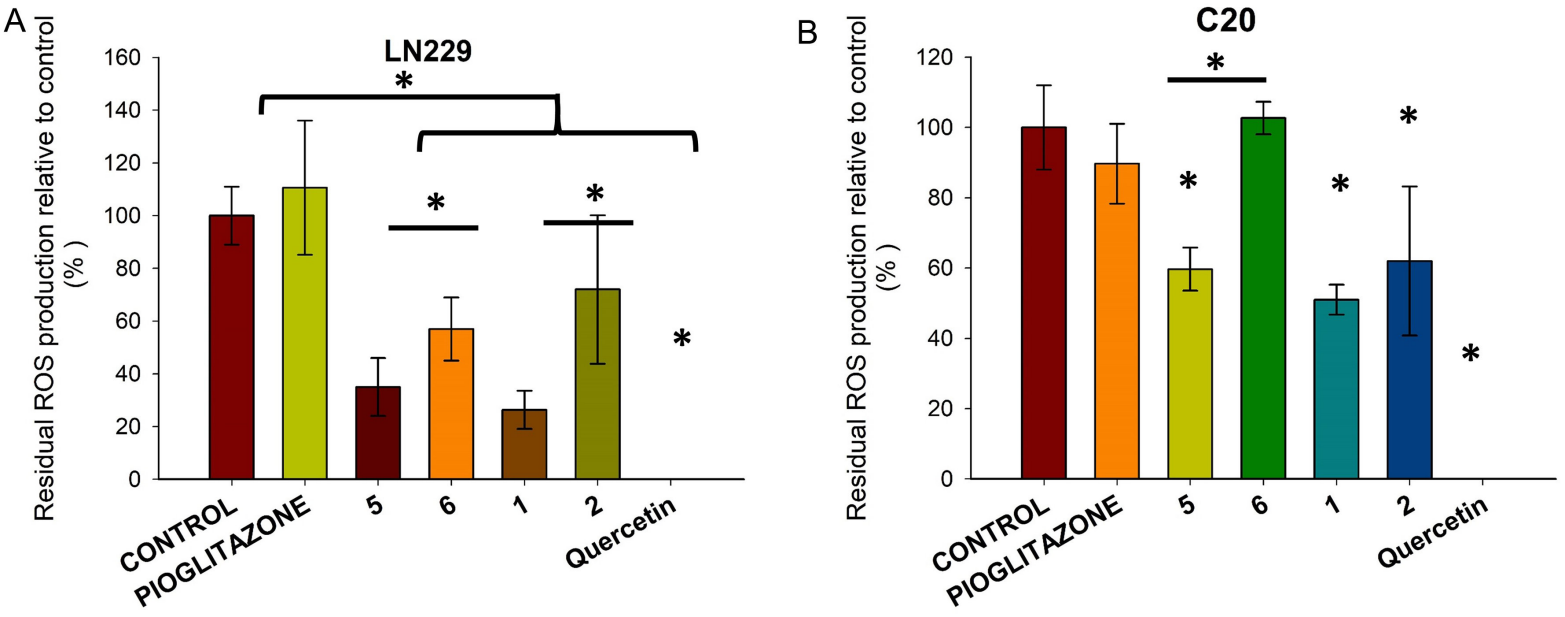
Evaluation of compounds **1**, **2**, **5**, and **6** on the ROS production induced by Antimycin A (AA, 4 µM) in LN229 (A) and C20 (B) cells. Quercetin and pioglitazone were also tested as standard ROS scavenger and reference inhibitor, respectively. After the pre‐loading with DCF‐DA, cells were treated with Quercetin (1 µM) or compounds (5 µM), in the presence or absence of Antimycin A. The ROS generation was detected for 30 min. The difference between the detected If/min of the tested sample in the presence and in the absence of AA was calculated, and the results are expressed as a percentage relative to the control sample (untreated with any compound, but only with an equivalent amount of solvent, DMSO). The shown data represent the mean ± SD of at least three independent experiments and eight sample replicates. The control samples (untreated with compound) versus the other samples (in the presence of the various compounds) were compared using Student’s t‐test; *p < 0.05.

Based on these results, the capability to neutralize ROS was further assessed in the more complex cellular system (LN229 and C20 cell lines). The ROS production in cells was induced by co‐treatment with Antimycin A, an inhibitor of complex III of the mitochondrial electron transport chain. As shown in Figure 6, compounds **1** and **5** showed a good capability to mitigate ROS levels induced by Antimycin A in both cell lines and are more effective than their analogs lacking the disulfide moiety. Unlike the in vitro assay, compounds **2** and **6** also reduced the DCF oxidation rate, even if with less extent than **1** and **5**. This behavior suggests that the disulfide moiety is important but not essential for this scavenging effect, and other mechanisms or cellular targets are involved in accounting for **2** and **6**’s antioxidant activities. However, it is worth underlining that the flavonoid quercetin remained the most active in this respect (no ROS detected) also in the cellular environment. Notably, compounds **1** and **5** demonstrated a great ROS scavenging effect compared to the parent compound pioglitazone, which resulted completely devoid of any antioxidant properties in these experimental conditions.

**FIGURE 7:**
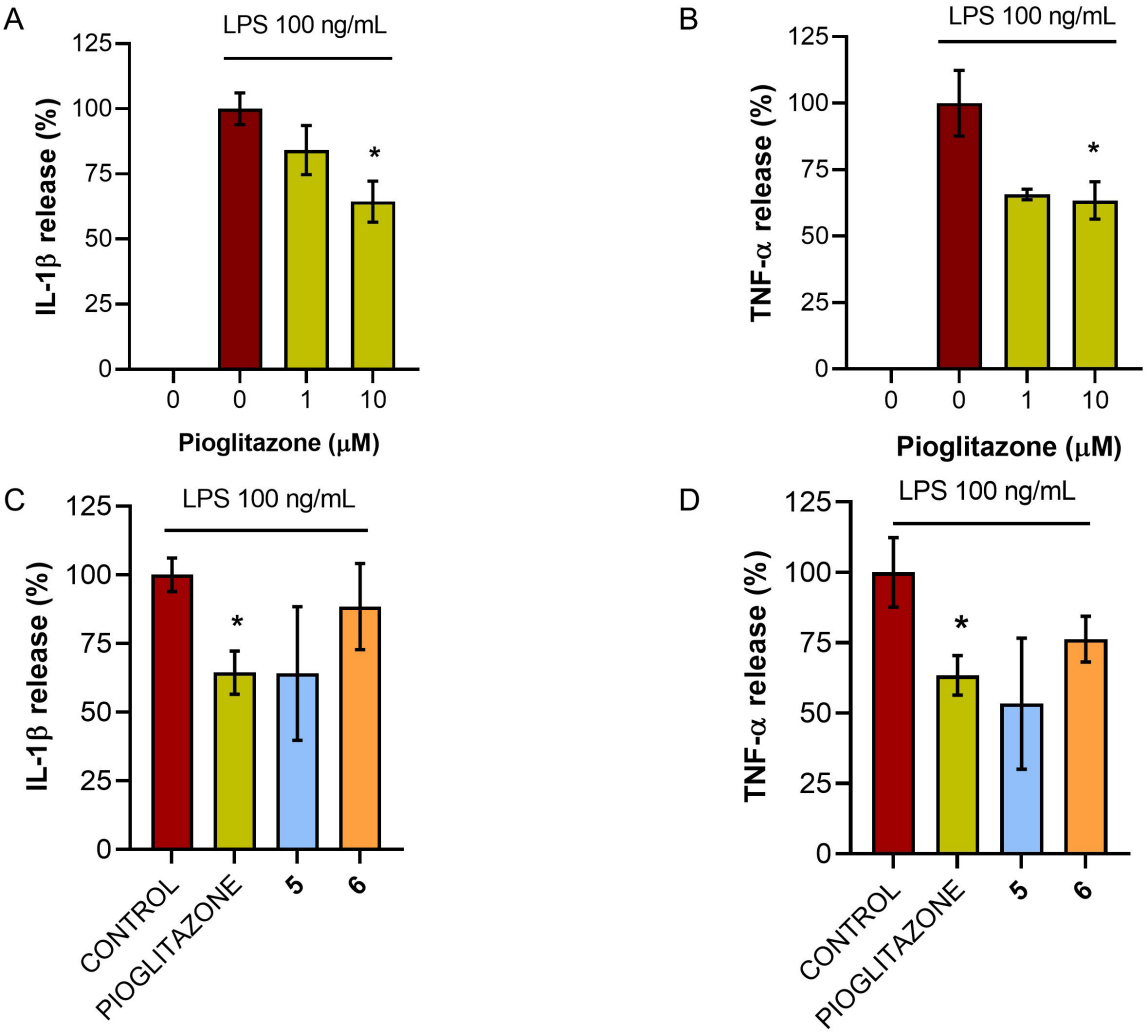
Evaluation of compounds **5**, **6**, and pioglitazone on pro‐inflammatory cytokine production in astrocyte‐enriched cultures. Cells were cultured in medium containing 10% serum, which was replaced with serum‐free medium before 1 h of pretreatment with pioglitazone (1–10 μM), **5**, or **6** (100 μM), followed by stimulation with LPS (100 ng/mL) for an additional 24 h. Supernatants were collected and analyzed for IL‐1β (A,C) and TNF‐α (B,D) release. Results are expressed as a percentage of cytokine release relative to LPS‐stimulated astrocytes (brown bars). Data are reported as mean ± SEM of 3 independent experiments. *p *< *0.05, versus LPS‐stimulated cells. One‐way ANOVA followed by Holm–Sidak’s multiple comparison test for panels A and B; Student’s *t*‐test versus LPS‐treated (control) cells for panels C and D.

Finally, the most effective compounds (**5**, **6**, and the reference compound pioglitazone) were evaluated for their ability to reduce the inflammatory response in astrocyte‐enriched cultures. Cells were pretreated for 1 h with three compounds before exposure to the endotoxin LPS (100 ng/mL) for 24 h to induce an inflammatory response, which was quantified by ELISA measurement of the pro‐inflammatory cytokines interleukin (IL)‐1β and tumor necrosis factor (TNF)‐α released into the culture medium. Unstimulated cells released low or undetectable amounts of IL‐1β and TNF‐α. LPS treatment markedly induced release of both cytokines (set to 100%), and this effect was significantly reduced by 10 μM pioglitazone to about 64% (Figure 7A and B). Under these experimental conditions, compound **5** appeared to be as effective as pioglitazone only at 100 µM concentration (Figure 7C and D); however, the effect did not reach statistical significance due to the high variability of the response. To note, this one‐order of magnitude discrepancy between compound **5** and pioglitazone’s anti‐inflammatory profiles agrees with the decrease in potency of **5** toward the two targets of interest with respect to the parent compound. In contrast, **6** failed to inhibit LPS‐induced release of IL‐1β (Figure 7C) and was also less effective in reducing TNF‐α release (Figure 7D). To note, none of the compounds affected cell viability at the concentrations used (Figure S6), indicating that the reduction in cytokine release observed with pioglitazone and **5** was not due to cytotoxic effects.

## 3 Conclusion

In recent years, many glitazones have been tested in clinical trials as potential therapeutics for different neurodegenerative diseases on the basis of promising preclinical neuroprotective properties resulting from MAO‐B and PPARγ modulation. Among these, pioglitazone outperformed at the preclinical level, albeit leading to a lack of efficacy at the clinical stage as putative AD and PD treatments. Starting from these premises, we and others focused on developing new derivatives with the aim to broaden the neuroprotective portfolio of the glitazone family [12, 42, 43]. In this work, a new small series of compounds was reported, featuring different disulfide‐bearing tails attached to the 5‐benzyl‐2,4‐thiazolidinedione head, looking for the potential covalent modulation of the two targets of interest. Compound **5** emerged as the most promising glitazone derivative of the series, characterized by a peculiar biological profile. It acted as a low micromolar selective and competitive MAO‐B inhibitor (Ki = 1.8 µM in vitro and IC_50_ = 0.8 µM in rat astrocyte lysate) and as a selective PPARγ agonist (EC_50_ = 14.6 µM and Emax = 75%), featuring a very slow dissociation rate. In this regard, computational investigations supported the feasibility of a potential covalent interaction between the disulfide bridge of **5** and Cys285 of PPARγ, while in MAO‐B the cysteine of interest finds itself too far away from this reactive moiety, thereby justifying the different mechanism of action toward the two targets. However, additional investigations are needed to determine its exact mechanism of action. At the cellular level, it showed no cytotoxicity in several cell lines, paired with a significant antioxidant and mild anti‐inflammatory effect. Particularly, pioglitazone maintained superior efficacy in reducing pro‐inflammatory cytokine expression, while the inserted disulfide tails enriched the biological portfolio of new derivatives with ROS scavenging properties with respect to the parent compound. Despite further insights to fully elucidate the biological profile of newly reported compounds are still ongoing, this work can represent the starting point for the future development of pioglitazone‐derived neuroprotective agents bearing a covalent mechanism of action.

## 4 Experimental Section

### 4.1 Chemistry

Chemical reagents were purchased from Merck, TCI and Fluorochem. Nuclear magnetic resonance spectra (NMR) were recorded at 400 or 600 MHz for ^1^H and 100.8 MHz for ^13^C on Varian MR400 or Bruker Ascend 600 spectrometer in CDCl_3_, DMSO‐*d*_6_ or CD_3_OD as solvents. Chemical shifts (d) are given in ppm from tetramethylsilane (TMS) with the solvent resonance as internal standard (CDCl_3_: δ 7.26, DMSO‐*d*_6_: δ 2.50, CD_3_OD: δ 3.31 for ^1^H NMR and CDCl_3_: δ 77.16, DMSO‐*d*_6_: δ 39.52, CD_3_OD: δ 49.00 for ^13^C NMR). For ^1^H NMR, data are reported as follows: chemical shift, multiplicity (s = singlet, d = doublet, dd = double of doublets, t = triplet, q = quartet, m = multiplet, *p *= pentet, dt = doublet of triplets, td = triplet of doublets, tt = triplet of triplets, qd = quartet of doublets, br s = broad singlet), coupling constants (Hz) and integration. Chromatographic separations were performed on silica gel columns through flash or gravity column (Kieselgel 40, 0.040–0.063 mm; Merck) chromatography. Reactions were followed by thin‐layer chromatography (TLC) on Merck (0.25 mm) glass‐packed pre‐coated silica gel plates (60 F254) that were visualized in an iodine chamber, or with a UV lamp, KMnO_4_, or bromocresol green. Final compounds’ mass spectra were recorded on a Waters ACQUITY ARC UHPLC/MS system (Milan, Italy). All final compounds were pure > 95% as determined via UHPLC/MS analyses run on a Waters ACQUITY ARC UHPLC/MS system, consisting of a QDa mass spectrometer equipped with an electrospray ionization (ESI) interface and a 2489 UV/Vis detector. The detected wavelengths (*λ*) were 254 and 365 nm. The analyses were performed on an XBridge BEH C18 column (10 mm × 2.1 mm i.d., particle size 2.5 µm) with an XBridge BEH C18 VanGuard Cartridge precolumn (5 mm × 2.1 mm i.d., particle size 1.8 µm). The mobile phases were H_2_O (0.1% formic acid) (A) and MeCN (0.1% formic acid) (B). ESI in positive and negative modes was applied in the mass scan range 50–1200 Da. Method and gradients used were the following: hydrophilic gradient: 0–0.5 min, 5% B; 0.5–1.5 min, 25% B; 1.5–2 min, 25% B; 2–3.5 min, 70% B; 3.5–3.9 min, 70% B; 3.90–4 min, 5% B; 4–5.73 min, 5% B; generic gradient: 0–0.78 min, 20% B; 0.78–2.87 min, 20%–95% B; 2.87–3.54 min, 95% B; 3.54–3.65 min, 95%–20% B; 3.65–5.73, 20% B. Flow rate: 0.8 mL/min. Generic gradient was used for compounds **5** and **6**, while compounds **1–4** required a hydrophilic gradient. High‐resolution mass spectra (HRMS) were recorded with an Xevo G2XS QTof apparatus with ESI in positive and negative mode. Compounds were named based on the naming algorithm developed by CambridgeSoft and used in ChemBioDraw Ultra (PerkinElmer, Milan, Italy, version 25.5.0).

#### 4.1.1 5‐(4‐Isothiocyanatobenzyl)thiazolidine‐2,4‐dione (8)

To a solution of **7** (mg, mmol) in DMF (2 mL) was slowly added a solution of TCDI (mg, mmol) in DMF (1 mL), and the reaction mixture was stirred at room temperature for 2 h. After completion, checked by TLC, the solvent was evaporated under reduced pressure, and the obtained crude was purified through column chromatography using mobile phase petroleum ether/ethyl acetate (6/4). Compound **8** was obtained as yellow powder (mg, %). ^1^H NMR (400 MHz, DMSO‐*d*_6_) δ 12.03 (br s, 1H), 7.38 (d, *J* = 8.6 Hz, 2H), 7.31 (d, *J* = 8.6 Hz, 2H), 4.92 (dd, *J* ^1^ = 4.6 Hz, *J* ^2^ = 8.8 Hz, 1H), 3.40–3.36 (m, 1H), 3.16 (dd, *J* ^1^ = 8.8 Hz, *J* ^2^ = 14.1 Hz, 1H). ^13^C NMR (100.6 MHz, DMSO‐*d*_6_) δ 175.98, 171.91, 137.34, 131.25, 129.30, 126.35, 123.96, 52.63, 36.98.

#### 4.1.2 General Procedure for Thiourea Formation

To a solution of the appropriate *N*‐Boc‐diamine (1 eq) and Et_3_N (1 eq) in DMF (1 mL) was added a solution of **7** (1 eq) dropwise in DMF (1 mL), and the reaction mixture was stirred at room temperature for 2 h. After completion, checked by TLC, the solvent was evaporated under reduced pressure, and the resulting crude was purified through column chromatography to obtain the pure product.

#### 4.1.3 *tert*‐Butyl (2‐((2‐(3‐(4‐((2,4‐dioxothiazolidin‐5‐yl)methyl)phenyl)thioureido)ethyl)disulfaneyl)ethyl)carbamate (9)

Compound **9** was obtained following the general procedure for thiourea formation using *N*‐Boc‐cystamine (134 mg, 0.53 mmol). Compound was eluted with DCM/MeOH/30% NH_4_OH_(aq)_ (9/1/0.1), which afforded **9** as a pale‐yellow oil (200 mg, 73%). ^1^H NMR (400 MHz, CDCl_3_) δ 10.54 (br s, 1H), 8.65 (br s, 1H), 7.20–7.13 (m, 4H), 6.94 (br s, 1 H), 5.24 (br s, 1H), 4.47–4.43 (m, 1H), 3.85–3.81 (m, 2H), 3.37–3.29 (m, 3H), 3.07–3.01 (m, 1H), 2.88–2.85 (m, 2H), 2.67–2.64 (m, 2H), 1.31 (s, 9H). ^13^C NMR (100.6 MHz, CDCl_3_) δ 180.45, 175.04, 171.29, 156.15, 136.41, 134.10, 130.51, 124.77, 79.80, 53.05, 43.41, 39.34, 37.84, 37.23, 31.66, 28.36.

#### 4.1.4 *tert*‐Butyl (6‐(3‐(4‐((2,4‐dioxothiazolidin‐5‐yl)methyl)phenyl)thioureido)hexyl)carbamate (10)

Compound **10** was obtained following the general procedure for thiourea formation using *N*‐Boc‐1,6‐hexanediamine (80 mg, 0.36 mmol). Compound was eluted with DCM/MeOH/30% NH_4_OH_(aq)_ (9/1/0.1), which afforded **10** as a pale‐yellow oil (88 mg, 51%). ^1^H NMR (600 MHz, CDCl_3_) δ 10.29 (br s, 1H), 8.40 (br s, 1H), 7.18 (d, *J* = 8.2 Hz, 2H), 7.13 (d, *J* = 8.2 Hz, 2H), 6.31 (br s, 1H), 4.70 (br s, 1H), 4.48 (dd, *J* ^1^ = 4.3 Hz, *J* ^2^ = 8.9 Hz, 1H), 3.52–3.50 (m, 2H), 3.37–3.35 (m, 1H), 3.12 (dd, *J* ^1^ = 8.9 Hz, *J* ^2^ = 14.2 Hz), 3.01–2.98 (m, 2H), 1.52–1.49 (m, 2H), 1.39–1.35 (m, 11H), 1.26–1.23 (m, 4H). ^13^C NMR (100.6 MHz, CDCl_3_) δ 180.15, 174.93, 171.13, 156.32, 136.18, 134.29, 130.91, 124.97, 79.43, 52.94, 45.13, 40.33, 37.82, 29.81, 28.74, 28.43, 26.32, 26.19.

#### 4.1.5 General Procedure for Urea Formation

To a solution of compound **7** (1 eq), phenyl chloroformate (1 eq), and Et_3_N (1.3 eq) in THF (2 mL) was added a solution of appropriate *N*‐Boc‐diamine (1 eq) and Et_3_N (1 eq) in THF (1.5 mL), and the reaction mixture was stirred at room temperature for 24 h. After completion, checked by TLC, the solvent was evaporated under reduced pressure, and the resulting crude was purified through column chromatography using DCM/MeOH (9.8/0.2) as the mobile phase to obtain the pure product.

#### 4.1.6 *tert*‐Butyl (2‐((2‐(3‐(4‐((2,4‐dioxothiazolidin‐5‐yl)methyl)phenyl)ureido)ethyl)disulfaneyl)ethyl)carbamate (11)

Compound **11** was obtained following the general procedure for urea formation using *N*‐Boc‐cystamine (162 mg, 0.64 mmol), affording it as a pale‐yellow oil (70 mg, 22%). ^1^H NMR (600 MHz, DMSO‐*d*_6_) δ 12.00 (br s, 1H), 8.56 (br s, 1H), 7.32 (d, *J* = 8.5 Hz, 2H) 7.09 (d, *J* = 8.5 Hz, 2H), 6.99 (t, *J* = 5.8 Hz, 1H), 6.31 (t, *J* = 5.8 Hz, 1H), 4.85 (dd, *J* ^1^ = 4.2 Hz, *J* ^2^ = 9.2 Hz, 1H), 3.40–3.36 (m, 2H), 3.29 (dd, *J* ^1^ = 4.2 Hz, *J* ^2^ = 14.2 Hz, 1H), 3.24–3.18 (m, 2H), 3.04 (dd, *J* ^1^ = 9.2 Hz, *J* ^2^ = 14.2 Hz, 1H), 2.82 (t, *J* = 6.6 Hz, 2H), 2.77 (t, *J* = 6.6 Hz, 2H), 1.38 (m, 9H). ^13^C NMR (100.6 MHz, DMSO‐*d*_6_) δ 176.24, 172.20, 156.00, 155.53, 139.81, 129.96, 129.67, 118.14, 78.26, 53.53, 49.07, 38.72, 38.45, 38.16, 37.03, 28.69.

#### 4.1.7 *tert*‐Butyl (6‐(3‐(4‐((2,4‐dioxothiazolidin‐5‐yl)methyl)phenyl)ureido)hexyl)carbamate (12)

Compound **12** was obtained following the general procedure for urea formation using *N*‐Boc‐1,6‐hexanediamine (195 mg, 0.90 mmol), affording it as a pale‐yellow oil (90 mg, 22%). ^1^H NMR (600 MHz, DMSO‐*d*_6_) δ 12.00 (br s, 1H), 8.36 (br s, 1H), 7.31 (d, *J* = 8.7 Hz, 2H), 7.08 (d, *J* = 8.7 Hz, 2H), 6.76 (br s, 1H), 6.10 (br s, 1H) 4.85 (dd, *J* ^1^ = 4.3 Hz, *J* ^2^ = 9.2 Hz, 1H), 3.29 (dd, *J* ^1^ = 4.3 Hz, *J* ^2^ = 14.1 Hz, 1H), 3.07–3.00 (m, 3H), 2.92–2.88 (m, 2H), 1.42–1.36 (m, 13H), 1.27–1.23 (m, 4H). ^13^C NMR (100.6 MHz, DMSO‐*d*_6_) δ 176.32, 172.26, 156.05, 155.63, 140.02, 129.93, 129.45, 118.00, 77.76, 53.58, 39.43, 37.05, 30.21, 29.94, 28.75, 26.56, 26.51.

#### 4.1.8 General Procedure for Boc Deprotection

To a solution of appropriate Boc‐protected amine (1 eq) in DCM (1 mL) at −20 °C was added HCl 4 M in dioxane (1 mL) dropwise and left stirring at this temperature for 2 h. After solvent evaporation under reduced pressure, the obtained powder was filtered and washed with diethyl ether to achieve the pure product.

#### 4.1.9 1‐(2‐((2‐Aminoethyl)disulfaneyl)ethyl)‐3‐(4‐((2,4‐dioxothiazolidin‐5‐yl)methyl)phenyl)thiourea Hydrochloride Salt (1)

Compound **1** was obtained following the general procedure for Boc deprotection using compound **9** (100 mg, 0.19 mmol), affording it as a white powder (80 mg, 91%). ^1^H NMR (600 MHz, DMSO‐*d*_6_) δ 12.07 (br s, 1H), 8.29 (br s, 2H), 8.18 (br s, 1H), 7.44 (d, *J* = 8.4 Hz, 2H), 7.18 (d, *J* = 8.4 Hz, 2H), 7.13 (br s, 1H), 4.91 (dd, *J* ^1^ = 4.3 Hz, *J* ^2^ = 9.4 Hz, 1H), 3.80–3.77 (m, 1H), 3.37–3.34 (dd, *J* ^1^ = 4.3 Hz, *J* ^2^ = 14.1 Hz, 1H), 3.13–3.08 (m, 4H), 3.01–2.97 (m, 4H). ^13^C NMR (100.6 MHz, DMSO‐*d*_6_) δ 180.97, 176.13, 172.10, 138.79, 132.88, 129.76, 123.03, 53.23, 43.06, 38.39, 37.15, 37.01, 34.57. MS [ESI+] *m/z*: 417.15 [M+H]^+^. HRMS (ESI): *m/z* calculated for C_15_H_20_N_4_O_2_S_4_ [M+H]^+^ 417.05421, found 417.05431.

#### 4.1.10 1‐(6‐Aminohexyl)‐3‐(4‐((2,4‐dioxothiazolidin‐5‐yl)methyl)phenyl)thiourea Hydrochloride Salt (2)

Compound **2** was obtained following the general procedure for Boc deprotection using compound **10** (88 mg, 0.18 mmol), affording it as a white powder (65 mg, 87%). ^1^H NMR (400 MHz, DMSO‐*d*_6_) δ 12.04 (br s, 1H), 9.99 (br s, 1H), 8.18 (br s, 1H), 7.90 (br s, 2H), 7.44 (d, *J* = 8.4 Hz, 2H), 7.14 (d, *J* = 8.4 Hz, 2H), 4.88 (dd, *J* ^1^ = 4.3 Hz, *J* ^2^ = 9.3 Hz, 1H), 3.43–3.41 (m, 2H), 3.33 (dd, *J* ^1^ = 4.3 Hz, *J* ^2^ = 14.1 Hz, 1H), 3.04 (dd, *J* ^1^ = 9.3 Hz, *J* ^2^ = 14.1 Hz, 1H), 2.78–2.70 (m, 2H), 1.58–1.47 (m, 4H), 1.34–1.29 (m, 4H). ^13^C NMR (100.6 MHz, DMSO‐*d*_6_) δ 180.76, 176.11, 172.08, 139.18, 132.37, 129.59, 122.61, 53.26, 43.85, 39.11, 37.13, 28.68, 27.32, 26.34, 25.98. MS [ESI+] *m/z*: 381.25 [M+H]^+^. HRMS (ESI): *m/z* calculated for C_17_H_24_N_4_O_2_S_2_ [M+H]^+^ 381.14135, found 381.14235.

#### 4.1.11 1‐(2‐((2‐Aminoethyl)disulfaneyl)ethyl)‐3‐(4‐((2,4‐dioxothiazolidin‐5‐yl)methyl)phenyl)urea Hydrochloride Salt (3)

Compound **3** was obtained following the general procedure for Boc deprotection using compound **11** (70 mg, 0.14 mmol), affording it as a white powder (47 mg, 77%). ^1^H NMR (600 MHz, DMSO‐*d*_6_) δ 12.07 (br s, 1 H), 9.01 (br s, 1H), 8.19 (br s, 2H), 7.39 (d, *J* = 8.5 Hz, 2H), 7.14 (d, *J* = 8.5 Hz, 2H), 6.69 (br s, 1H), 4.91 (dd, *J* ^1^ = 4.3 Hz, *J* ^2^ = 9.1 Hz, 1H), 3.46–3.43 (m, 2H), 3.34 (dd, *J* ^1^ = 4.3 Hz, *J* ^2^ = 14.1 Hz, 1H), 3.18–3.15 (m, 2H), 3.08 (dd, *J* ^1^ = 9.1 Hz, *J* ^2^ = 14.1 Hz, 1H), 3.03 (t, *J* = 6.5 Hz, 2H), 2.91 (t, *J* = 6.5 Hz, 2H). ^13^C NMR (100.6 MHz, DMSO‐*d*_6_) δ 176.18, 172.17, 155.70, 139.91, 129.95, 129.57, 118.01, 53.51, 38.63, 38.61, 38.42, 37.02, 34.61. MS [ESI+] *m/z*: 401.25 [M+H]^+^. HRMS (ESI): *m/z* calculated for C_15_H_20_N_4_O_3_S_3_ [M+H]^+^ 401.07700, found 401.07680.

#### 4.1.12 1‐(6‐Aminohexyl)‐3‐(4‐((2,4‐dioxothiazolidin‐5‐yl)methyl)phenyl)urea Hydrochloride Salt (4)

Compound **4** was obtained following the general procedure for Boc deprotection using compound **12** (90 mg, 0.19 mmol), affording it as a white powder (61 mg, 80%). ^1^H NMR (600 MHz, DMSO‐*d*_6_) δ 12.01 (br s, 1H), 8.83 (br s, 1H), 7.92 (br s, 2H), 7.32 (d, *J* = 8.5 Hz, 2H), 7.07 (d, *J* = 8.5 Hz, 2H), 6.44 (br s, 1H), 4.85 (dd, *J* ^1^ = 4.3 Hz, *J* ^2^ = 9.1 Hz, 1H), 3.28 (dd, *J* ^1^ = 4.3 Hz, *J* ^2^ = 14.1 Hz, 1H), 3.06 (t, *J* = 6.8 Hz, 2H), 3.02 (dd, *J* ^1^ = 9.1 Hz, *J* ^2^ = 14.1 Hz, 1H), 2.79–2.73 (m, 2H), 1.58–1.53 (m, 2H), 1.44–1.39 (m, 2H), 1.34–1.29 (m, 4H). ^13^C NMR (100.6 MHz, DMSO‐*d*_6_) δ 176.19, 172.18, 155.83, 140.19, 129.90, 129.24, 117.82, 53.55, 39.25, 39.14, 37.03, 30.00, 27.36, 26.29, 25.98. MS [ESI+] *m/z*: 365.33 [M+H]^+^. HRMS (ESI): *m/z* calculated for C_17_H_24_N_4_O_3_S [M+H]^+^ 365.16416, found 365.16446.

#### 4.1.13 General Procedure for Bis Coupling

To a solution of appropriate dicarboxylic acid (1 eq) in DMF (3 mL) at 0 °C HOBt (2.5 eq) and EDC (2.5 eq) were added. Once checked by TLC the complete conversion of the starting reagent, compound **7** (2 eq), was checked by TLC, the reaction mixture was stirred for 4 h at room temperature. After solvent evaporation under reduced pressure, the crude product was purified through column chromatography to achieve the pure product.

#### 4.1.14 2,2ʹ‐Disulfanediylbis(*N*‐(4‐((2,4‐dioxothiazolidin‐5‐yl)methyl)phenyl)acetamide) (5)

Compound **5** was obtained following the general procedure for bis coupling using dithiodiglycolic acid (98 mg, 0.54 mmol). Compound was eluted with DCM/MeOH (9.7/0.3), affording it as a pale‐yellow oil (110 mg, 34%). ^1^H NMR (600 MHz, DMSO‐*d*_6_) δ 12.04 (br s, 2H), 10.19 (br s, 2H), 7.53 (d, *J* = 8.5 Hz, 4H), 7.19 (d, *J* = 8.5 Hz, 4H), 4.88 (dd, *J* ^1^ = 4.3 Hz, *J* ^2^ = 9.2 Hz, 2H), 3.73 (s, 4H), 3.35 −3.32 (m, 2H), 3.08 (dd, *J* ^1^ = 9.2 Hz, *J* ^2^ = 14.1 Hz, 2H). ^13^C NMR (100.6 MHz, DMSO‐*d*_6_) δ 176.25, 172.20, 167.13, 138.16, 132.38, 130.11, 119.71, 53.34, 43.61, 37.11. MS [ESI+] *m/z*: 591.18 [M+H]^+^. HRMS (ESI): *m/z* calculated for C_24_H_22_N_4_O_6_S_4_ [M‐H]^‐^ 589.03498, found 589.03468.

#### 4.1.15 *N* ^1^,*N* ^6^‐Bis(4‐((2,4‐dioxothiazolidin‐5‐yl)methyl)phenyl)adipamide (6)

Compound **6** was obtained following the general procedure for bis coupling using adipic acid (53 mg, 0.36 mmol). Compound was eluted with DCM/MeOH (9.5/0.5), affording it as a pale‐yellow oil (44 mg, 22%). ^1^H NMR (600 MHz, DMSO‐*d*_6_) δ 12.05 (br s, 2H), 9.90 (br s, 2H), 7.52 (d, *J* = 8.5 Hz, 4H), 7.15 (d, *J* = 8.5 Hz, 4H) 4.87 (dd, *J* ^1^ = 4.3 Hz, *J* ^2^ = 9.1 Hz, 2H), 3.33 −3.30 (m, 2H), 3.06 (dd, *J* ^1^ = 9.1 Hz, *J* ^2^ = 14.2 Hz, 2H), 2.34–2.31 (m, 2H), 1.62–1.61 (m, 2H). ^13^C NMR (100.6 MHz, DMSO‐*d*_6_) δ 176.16, 172.15, 171.52, 138.73, 131.62, 129.97, 119.48, 53.35, 37.06, 36.71, 25.34. MS [ESI+] *m/z*: 555.37 [M+H]^+^. HRMS (ESI): *m/z* calculated for C_26_H_26_N_4_O_6_S_2_ [M‐H]^‐^ 553.12212, found 553.12172.

### 4.2 Computational Studies

#### 4.2.1 Protein and Ligand Preparation

The X‐ray crystal structures of human MAO‐B (PDB code: 4A79) and human PPARγ (PDB code: 5Y2O) were retrieved from the Protein Data Bank. Protein structures were prepared using the Molecular Operating Environment (MOE) suite (version 2024.0601) [44]. Water molecules and co‐crystallized ligands were removed, whereas the FAD cofactor in *h*MAO‐B was retained as part of the catalytic pocket. Hydrogen atoms were added to the protein structures using standard geometries. To relieve steric clashes and minimize contacts between hydrogens, the structures were subjected to energy minimization using the Amber99 force field until the root‐mean‐square (RMS) gradient fell below 0.1 kcal mol^−1 ^Å^−1^, keeping the heavy atoms fixed at their crystallographic positions. The selected compounds (**1**, **5**, and **6**) were built using the MOE builder module, considering both their intact states and, for **5**, the reduced “cleaved” state bearing a free thiol group. Gasteiger partial charges were assigned prior to the docking simulations.

#### 4.2.2 Docking at *h*MAO‐B and *h*PPARγ

Standard noncovalent docking simulations for *h*MAO‐B were performed using the MOE Dock module. The binding site was defined around the FAD cofactor and the established catalytic pocket. Ligand placement was executed using the Triangle Matcher algorithm, and initial poses were ranked according to the London dG scoring function. The receptor was subjected to an Induced Fit refinement protocol. The final binding affinity was estimated using X‐Score, an empirical scoring function that incorporates terms for van der Waals interactions, hydrogen bonding, deformation penalties, and hydrophobic effects. To ensure the robustness and reproducibility of the docking procedure, the noncovalent protocol was validated through an extensive redocking and cross‐docking campaign. A carefully selected panel of diverse reversible inhibitors was docked into the *h*MAO‐B reference structure (PDB: 4A79). The test set included the native ligand pioglitazone, as well as safinamide, rasagiline, rosiglitazone, and isatin extracted from their respective high‐resolution complexes. The top‐ranked docking poses successfully reproduced the experimental crystallographic geometries, yielding an excellent average root‐mean‐square deviation (RMSD) of 1.62 ± 0.32 Å (Table 3), thereby confirming the reliability of the triangle matcher placement and London dG scoring functions.

To properly evaluate the mechanism of action of **5** at PPARγ, a covalent docking protocol was employed in parallel with standard docking simulations. This approach specifically modeled the formation of a disulfide bond between the free thiol of the “cleaved” molecule and residue Cys285. The MOE Covalent Dock protocol was utilized, setting the reaction type to “disulfide formation”. The reactive site was explicitly defined on the sulfur atom of Cys285. During the automated placement process, an alignment stub was generated based on the reactive pocket residues to accurately guide the connection of the ligand warhead. The conformational space of the ligand was thoroughly sampled by generating unique lower‐energy ring and chiral classes, followed by systematic rotamer generation. Each generated conformer was positioned within the binding pocket by aligning it to the reaction stub and further optimized through small rigid‐body translations and rotations. These initial complex poses were evaluated using the Alpha Score function, deliberately masking the atoms immediately proximate to the newly formed protein‐ligand connection to prevent artificial repulsive interactions. To accurately model the target accommodation of the covalent adduct, the generated complexes were subsequently subjected to an Induced Fit refinement protocol, allowing local flexibility of the binding pocket residues. The final optimized poses were ranked using the GBVI/WSA dG scoring function, which rigorously evaluates both the geometric quality and strain energy of the covalent linkage, as well as the complementary noncovalent interactions within the PPARγ binding pocket. The intact, noncleavable analog **6** was evaluated as a negative control for this covalent mechanism. Similarly, the docking protocols for PPARγ were rigorously validated using a multiset structural ensemble exclusively comprising agonist‐bound states. The standard noncovalent protocol was validated by cross‐docking canonical full agonists (pioglitazone, lobeglitazone, and rosiglitazone) into the reference 5Y2O structure, achieving an average RMSD of 1.30 ± 0.17 Å (Table 4). Crucially, to validate the covalent docking parameters without relying on antagonist structures, we successfully reproduced the binding mode of 15‐deoxy^Δ12,14^‐prostaglandin J2 (PDB: 2ZK1), a well‐characterized endogenous agonist that natively forms a covalent adduct with Cys285. The algorithm accurately recreated the native covalent linkage and noncovalent interactions within the binding pocket with an RMSD of 1.52 Å (Table 4), fully validating the selected parameters for evaluating the covalent engagement of compound **5**.

**TABLE 4:** Comprehensive validation of the docking protocols at *h*MAO‐B (PDB: 4A79) and *h*PPARγ (PDB: 5Y2O).

Target	Protocol	Ligand	Native PDB ID	RMSD, Å
*h*MAO‐B	Noncovalent	Pioglitazone (R)	4A79	1.19
		Safinamide (C)	2V5Z	1.58
		Rosiglitazone (C)	4A7A	1.77
		Isatin (C)	1OJ9	1.94
		Average		1.62 ± 0.32
*h*PPARγ	Noncovalent	Pioglitazone (R)	5Y2O	1.11
		Lobeglitazone (C)	5Y2T	1.34
		Rosiglitazone (C)	7AWC	1.44
		Average		1.30 ± 0.17
	Covalent	15d‐PGJ2 (C)	2ZK1	1.52

*Note*: The noncovalent and covalent docking procedures were rigorously validated via redocking (R) and cross‐docking (C) approaches using a multiset structural ensemble of native high‐resolution complexes.

### 4.3 Biological Evaluation Assay

#### 4.3.1 Monoamine Oxidase Activity Methods

Unless otherwise specified, all chemicals (analytical grade) and enzymes were from Merck (Milan, Italy). All compounds and pio‐glitazone were dissolved in dimethyl sulfoxide (DMSO) and used at a final concentration of 0.2% DMSO.

Monoamine oxidase activity of human recombinant enzymes (human recombinant MAO‐A and MAO‐B expressed in baculovirus‐infected BT1 cells) was determined by detecting the production of H_2_O_2_, one of the products of the reaction catalyzed by MAOs. This assay was carried out by using a peroxidase‐coupled continuous assay, which measures the H_2_O_2_ by using the Amplex Red reagent (10‐acetyl‐(3,7)‐dihydroxyphenoxazine) as a fluorogenic substrate for horseradish peroxidase to produce red‐fluorescent resorufin [45].

All experiments were carried out in 0.1 M potassium phosphate buffer, 1 mM ethylenediaminetetraacetic acid (EDTA), pH 7.4, and at 37 °C. The assay buffer solution (final volume of 800 µL for the assays carried out with the Cary–Eclipse fluorimeter or 200 µL for the assays carried out with the Spark Microplate Reader) contains Amplex Red (100 µM) and horseradish peroxidase type IV‐A (5 UmL^−1^), BZA or *p*‐Tyr as substrates for MAO‐B and MAO‐A, respectively. The rate of reactions was determined by measuring the increase in fluorescence intensity (*λ*_exc_ = 563 nm and *λ*_em_ = 586 nm) in one unit of time (IF/s, in arbitrary units, a.u.); the H_2_O_2_ production rate was calculated from the increase in fluorescence intensity, by means of calibration curves obtained by standard solutions of H_2_O_2_. Since at high tyramine concentrations the fluorescence intensity of resorufin decreases, the reaction rates of MAO‐A were calculated using specific calibration curves built for each concentration of the substrate used for the assay. No significant interference of the various compounds on fluorescence intensity (of calibration curves) was observed. If not different reported, hr‐MAO A and hr‐MAO B concentrations in the assay solutions were 2.5 and 5 µg/mL, respectively.

The assay of MAO activity in cell lysates was performed by measuring the formation of 4‐hydroxyquinoline as reaction product of MAO activity in the presence of kynuramine (Kyn) as substrate [46].

Briefly: the enzyme assay solution (200 µL final volume in buffer solution) contains the tested compound, Kynuramine substrate (20 µM, that is about at its *K*_M_ value, *K*_M_ = 21 ± 4 µM), and astrocyte lysates (about 75 µg/mL of final protein concentration) added last to the solution to start the enzymatic reaction. The enzymatic reaction was run for 30 min at 37 °C (in the dark). Then, the reaction was stopped by addition of 2 M NaOH (80 µL) and 480 µL of distilled water. Kynuramine deaminated by MAO spontaneously cyclizes to give 4‐hydroxyquinoline, the amount of which was determined by the fluorescence intensity of the peak of its emission spectra (*λ*_exc_ = 330 nm and *λ*_em_ = 330–530 nm, *λ*_em_,_peak_ = 380 nm), using a specific calibration curve built with the standard 4‐hydroxyquinoline. The specific activity of lysates determined at a saturating concentration of substrate (Kyn = 200 µM) was (As) = 2.1 ± 0.5 nmol_4HQ _min^−1 ^mg _protein_^−1^.

The presence of the MAO‐B isoform in rat astrocyte lysates was verified by using safinamide, the well‐known reversible and specific MAO‐B inhibitor: 35% ± 4% of residual MAO‐A activity was found, using Kyn at 20 µM and two different safinamide concentrations (0.2 and 2 µM), indicating that 65% of the total MAO activity is of the MAO‐B isoform.

For each type of kinetic experiment, a control sample (in the absence of the inhibitor) and a blank sample (in the absence of the enzyme) were also run under the same experimental conditions. The IF of the blank was subtracted from all the samples, before calculating the velocity (IF/s).

The reversibility of the inhibition of compound **5** was evaluated by extensive dialysis. The enzyme‐inhibitor complex, obtained by the addition of 200 µM compound **5** or Selegiline to *h*MAO‐B (65 µg/mL), was incubated for 20 min (in 0.1 M potassium phosphate buffer, 1 mM EDTA, pH 7.4, at 37 °C) before being subjected to extensive dialysis overnight in potassium phosphate 0.1 M, pH 7.2, at 4 °C. A control sample containing *h*MAO‐B was run simultaneously, under the same experimental conditions. The MAO activity assay on the recovered enzyme solution was carried out using 300 µM kynuramine as substrate.

A Cary‐Eclipse fluorimeter (Varian Inc., Palo Alto, CA, USA) or a Spark Multimode Microplate Reader (TECAN Italia, s.r.l.) were used for fluorometric measurements.

*Kinetic and statistical analysis*. The *V*_max_ and *K*_M_ values of *h*MAO‐A and *h*MAO‐B were determined in the presence of the different concentrations of the tested compounds. The mode of inhibition was determined by global fit analysis (GraphPad 9.0 software) of the experimental data (initial rate of reaction (V_0_) vs. substrate concentrations, at the various inhibitor concentrations), to fit equations for competitive, mixed, noncompetitive and uncompetitive inhibition models. The fit giving the highest *r* ^2^ value was selected for the calculation of inhibition constants (*K*_i_). The data in the figures are reported as Lineweaver–Burk plots (1/v vs. 1/S) to show the mode of inhibition of the tested compounds. Unless stated otherwise, the correlation coefficient for linear regression was 0.98 or greater.

The IC_50_ values were calculated by fitting the “inhibitor concentration versus response (three parameters)” equation to the experimental data (relative MAO activity with respect to the control sample, v/v_0_ vs. compound concentration) by using the GraphPad Prism 9.0 software.

The data are the result of at least three independent experiments, performed in triplicate. Results are expressed as the mean ± SD.

### 4.4 PPAR Assay

HepG2 human hepatoblastoma cells (RRID:CVCL_0027; Interlab Cell Line Collection, Genoa, Italy) were maintained under standard culture conditions in minimum essential medium (MEM) supplemented with 10% fetal bovine serum (FBS), 2 mM glutamine, 100 U/mL penicillin G, 100 μg/mL streptomycin, and 1x nonessential amino acid (NEAA) solution. Cells were grown at 37 °C in a humidified atmosphere with 5% CO_2_ and subcultured (1:5 to 1:10) twice a week.

Reference compounds, cell culture medium, and other reagents were obtained from Merck–Italy (Milan, Italy). Expression vectors encoding the chimeric receptor, comprising the yeast Gal4 DNA‐binding domain fused to the human PPARα, PPARγ, or PPARδ ligand‐binding domain, and the reporter plasmid for these Gal4 chimeric receptors (pGal5TKpGL3), which contains five repeats of the Gal4 response elements upstream of a minimal thymidine kinase promoter adjacent to the luciferase coding sequence, were previously described [47]. For transactivation assays, HepG2 cells were seeded in a 96‐well plate at a density of 3^.^10^5^ cells per well and transfected with “GenJet DNA in vitro transfection reagent” (SignaGen Laboratories, Frederick, MD, U.S.) according to the manufacturer’s guidelines. Transfection was performed using expression plasmids encoding the fusion protein Gal4‐PPARx‐LBD (20 ng), pGal5TKpGL3 (40 ng), and pCMVβgal (40 ng). The pCMVβgal plasmid, which expresses β‐galactosidase under the CMV promoter, was included as an internal control to normalize luciferase activity for transfection efficiency. Following a 24 h incubation, the culture medium was aspirated, and cells were treated in duplicate with the indicated ligands at concentrations ranging from 10 to 100 µM. The cells were then incubated for a further 20 h prior to cell lysis. For washout experiments, after 4 h preincubation with **5** or **6**, cells were rinsed twice with serum‐free medium prior to the addition of treatment‐containing medium. Cell lysates were subsequently analyzed for luciferase and β‐galactosidase activity using a multilabel plate reader (VICTOR3 V, PerkinElmer). Luciferase activity was normalized to β‐galactosidase activity and expressed as relative activity, compared with the response to the standard ligands, WY‐14643 (PPARα), pioglitazone (PPARγ), or L165,041 (PPARδ), which was set to 100%. These data were plotted in concentration‐response curves using GraphPad Prism 11 to derive the EC_50_ (µM) and Emax (%) values, which are reported as mean ± SEM of at least three independent experiments conducted in duplicate.

### 4.5 Assays for Antioxidant Activity

The in vitro assay used to evaluate the potential antioxidant activity of some compounds detects the oxidation of 2ʹ,7ʹ‐dichlorodihydrofluorescein (DCF) (in PBS buffer, phosphate‐buffered saline) induced by ABIP (2,2′‐azobis[2‐(2‐imidazolin‐2‐yl)propane]), a water‐soluble azo compound. ABIP is used as a radical initiator to produce a constant, controlled stream of carbon‐centered radicals, subsequently forming peroxyl radicals in the presence of oxygen. In detail, the oxidation of DCF (1 µM) caused by ABIP (62 µM) in PBS buffer (T = 37 °C) is continuously detected by monitoring the increase in fluorescence intensity (*λ*_ex_ = 488 nm; *λ*_em_ = 525 nm) with a Cary–Eclipse fluorimeter (Varian Inc., Palo Alto, CA, USA). The compound is then added, and the decrease in the rate of oxidation of DCF is measured. The residual DCF oxidation rate is compared to that in the absence of compounds. No direct effect on the DCF fluorescence was detected in the presence of compound and absence of ABIP.

### 4.6 Inhibition Growth Assay

LN229 human glioblastoma cells (ATCC CRL‐2611; RRID:CVCL_0393; LGC Standards Srl, Milan, Italy) were grown in DMEM (D2902, Merck) containing 3.7 g/L NaHCO_3_ (S5761, Merck), 3.5 g/L glucose (G6152, Merck), 100 U/mL penicillin, 100 μg/mL streptomycin, 0.25 μg/mL amphotericin B (A5955, Merck) and 5% (v/v) heat‐inactivated FBS (F7524, Merck). C20 cell line, a human immortalized microglia cell line, was originally generated by David Alvarez‐Carbonell et al. (Case Western Reserve University) [48] and kindly gifted by Prof. Barbara Costa (Dept. of Pharmacy, University of Pisa, Italy). These cells were grown in DMEM‐F12 (NB580049, Neo Biotech), supplemented with 600 μg/mL Neomycin (NB6401112, Neo Biotech), 100 U/mL penicillin, 100 μg/mL streptomycin (P0781, Merck), and 10% (v/v) heat‐inactivated FBS (F7524, Merck). The cells were maintained at 37 °C in a humidified atmosphere with 5% carbon dioxide in air.

To assay cell viability, 4 × 10^4^ LN229 or 5 × 10^4^ C20 cells were seeded into each well of a 24‐well cell culture plate and allowed to grow for 24 h. Then, test compounds were added at 20 µM to the complete medium, and cells were incubated for a further 48 h. Stock solutions of tested compounds were made in dimethyl sulfoxide at 20 mM concentration and then diluted with complete medium in such a way that the final amount of solvent did not exceed 0.4% (v/v). A Trypan blue assay was performed to determine cell viability.

### 4.7 Assay of ROS Production in Cells

3 × 10^3^ LN229 or 8 x 10^3^ C20 cells were seeded into P96 cell culture plates, incubated in complete growth medium at 37 °C in a 5% CO_2_ humidified atmosphere, and allowed to grow for 48 h. Then, the medium was replaced with PBS‐glucose solution (0.1 M NaCl, 2 mM KCl, 8 mM NaHPO_4_ 2H_2_O, 1.5 mM KH_2_PO_4_, 5 mM glucose) for LN‐229 and with DMEM culture medium for C20, both containing 10 μM 2ʹ,7ʹ‐dichlorodihydrofluorescein diacetate (DCFDA, D6883, Merck), and cells were incubated at 37 °C in the dark for 20 min. Cells were washed with PBS, and the PBS‐glucose solution (200 µL/well) containing the tested compounds (5 µM) or 1 µM quercetin (reference ROS scavenger) in the presence or absence of 4 µM antimycin A (ROS inducer) was added.

Fluorescence intensity (IF) was measured every 5 min for 30 min (*T* = 37 °C) by using a Spark Multimode Microplate Reader (TECAN) (*λ*_ex_ = 485 nm and *λ*_em_ = 535 nm). The autofluorescence samples (cells incubated without 2ʹ,7ʹ‐dichlorofluorescin diacetate) were subtracted from each sample, and the IF/min values were then calculated. Then, the difference between the IF/min of the tested sample in the presence and in the absence of AA was calculated, and results are expressed as a percentage relative to the control sample (untreated with any compound, but only with an equivalent amount of solvent, DMSO). The resulting data are expressed as the mean ± SD of at least three independent experiments and eight sample replicates. The control samples (untreated with compound) versus the other samples (in the presence of the various compounds) were compared using Student’s *t*‐test, using the GraphPad Prism software, version 9.0 (San Diego, CA, USA).

### 4.8 Rat Astrocyte Preparation and Assay

Sprague–Dawley rats (CD strain) were housed under controlled temperature and humidity, with *ad libitum* access to water and food, on a 12 h light/dark cycle. All animal‐related procedures were carried out in accordance with the Italian Ministry of Health guidelines (D.L. 26/2014) for the care and use of laboratory animals and were approved by the Institutional Animal Care and Use Committee (Organismo Preposto al Benessere Animale, OPBA) of the University of Padova and by the Italian Ministry of Health (Protocol #41451.N.WBR). Postnatal day 1 (PN1) rat pups of both sexes were rapidly decapitated to minimize suffering, discomfort, and stress. Astrocytes were isolated from mixed glial cell cultures prepared from the cerebral cortices of PN1 rat pups, as previously reported [49]. Briefly, once cultures reached confluence (typically 7 days after isolation), microglia were removed by shaking flasks on an orbital shaker (200 rpm for 1 h at 37 °C). The remaining cells were detached using a 0.25% trypsin/EDTA solution. The resulting cell suspension, representing a highly enriched astrocyte population [50], was collected and centrifuged (2400 rpm, 5 min). The cell pellet was re‐suspended in high‐glucose Dulbecco’s modified Eagle medium (DMEM) supplemented with 2 mM L‐glutamine, 10% heat‐inactivated FBS (Capricorn Scientific [Ebsdorfergrund, Germany]), 100 units/mL penicillin, 100 μg/mL streptomycin, and 50 μg/mL gentamicin. Cells were plated on poly‐D‐lysine‐coated plastic wells at a density of 1.20 × 10^4^ cells/cm^2^ and maintained at 37 °C in a humidified atmosphere containing 5% CO_2_/95% air.

#### 4.8.1 Cell Viability

The viability of astrocyte‐enriched cultures was assessed using the sulforhodamine B (SRB) assay [51, 52]. At the end of the treatments, cells were fixed with cold 10% trichloroacetic acid for 1 h at 4 °C. Cells were then stained with 0.4% SRB for 30 min at room temperature. After staining, the protein‐bound dye was solubilized in 10 mM Tris base solution. The absorbance was measured at 570 nm using a microplate reader. Absorbance of vehicle‐treated cultures was set as 100% cell viability.

#### 4.8.2 Cytokine Determination

Cells were pretreated for 1 h with increasing concentrations of pioglitazone (1–10 μM), **5**, and **6** (100 μM) and then stimulated with 100 ng/mL LPS (Ultrapure LPS‐EB from *Escherichia coli*, 0111:B4 strain, InvivoGen, Europe, Toulouse, France), dissolved in endotoxin‐free water (InvivoGen) for an additional 24 h. At the end of the incubation period, culture medium was collected, and IL‐1β and TNF‐α levels were measured using commercially available ELISA kits (Antigenix America, Huntington Station, NY, USA), according to the manufacturer’s instructions. The absorbance of each sample was measured at 450 nm using a microplate reader. Cytokine concentrations (pg/mL) in the medium were determined by reference to standard curves generated with known amounts of IL‐1β or TNF‐α. Control cultures contained the same concentration of vehicle.

#### 4.8.3 Statistical Analysis

All data represent the results of at least three independent experiments, each performed in triplicate. Results are expressed as means, with error bars representing the standard error of the mean (SEM). GraphPad Prism software, version 8.4 (San Diego, CA, USA) was used for statistical analysis. Differences between groups were calculated with one‐way ANOVA followed by Holm–Sidak’s *post hoc* test, or by the Student’s t‐test as detailed in the figure legend. Differences were considered statistically significant when *p *< 0.05.

### 4.9 Cell Lysates Preparation

Cells of the highly astrocyte‐enriched cell population (astrocytes cells) (approximately 2 × 10^6^) were seeded in P60 plates. After 4 days, cells were washed with phosphate buffer saline (PBS) (10 mM Na_2_HPO_4_, 1.8 mM KH_2_PO_4_, 2.7 mM KCl, 137 mM NaCl), trypsinized (Trypsin –EDTA 0.25%, 1 mL /dish) for 2 min, before adding the complete cell medium (DMEM‐high glucose concentration and 10% FBS). Cells were counted and then centrifuged (at 800 × g × 5 min). The pellet was resuspended, washed with PBS and 1 mM EDTA, and centrifuged (at 325 × g × 5 min, at *T* = 4 °C) for two times. The final cell pellet was frozen in liquid nitrogen until use. The lysis was performed rapidly, on ice, by adding about 1 mL of lysis buffer (20 mM Hepes (pH 7.4), 1 mM EDTA, and protease inhibitor cocktail (1:400 v/v) to 10 million thawed cell pellet. The lysate was immediately frozen in liquid nitrogen and stored at −80 °C until use. The protein content was measured by using the Bradford method with bovine serum albumin as the standard [53].

## Funding

This research was supported by institutional grants from the University of Bologna and University of Padova, BIRD 2025 (DALV_BIRD25_01 to L.D.V., M.L.D.P. and M.Z.), PRIN 2022 project “MitoMedChem for neurodegeneration” (Call for tender No. 104 published on 2.2.2022) funded by the Italian Ministry of University and Research (MUR) ‐ CUP C53D23004440006 to L.D.V. and M.L.D.P., European Union – Next‐Generation EU (“PNRR M4C2‐Investimento 1.4‐CN00000041”) to G.C.

## Conflicts of Interest

The authors declare no conflicts of interest.

## Supporting information

Supplemental Figures and Tables as well as NMR spectra and HPLC traces of final compounds are reported in Supporting Information. The authors have cited additional references within the Supporting Information [54].

## Data Availability

The data that support the findings of this study are available from the corresponding author upon reasonable request.
