# Supplementary material for: Exploring Disulfide Bridge as a Tool to Improve Pioglitazone’s Neuroprotective Potential: Toward the Development of Prolonged‐Acting MAO‐B/PPARγ Modulators
Source: ChemMedChem. 2026 Jul 29;21(15):e70405. doi: 10.1002/cmdc.70405 (PMC13420967; doi:10.1002/cmdc.70405)
Supplement: Supplementary file 1 — Supplemental Figures and Tables as well as NMR spectra and HPLC traces of final compounds are reported in Supporting Information. The authors have cited additional references within the Supporting Information [54]. [file CMDC-21-e70405-s001.pdf]

Exploring disulfide bridge as a tool to improve pioglitazone's neuroprotective potential: toward the development of prolonged-acting MAO-B/PPARγ modulators

Filippo Basagni[a],*, Maria Luisa Di Paolo[b], Antonio Laghezza[c], Giorgio Cozza[b], Francesco Piazzola[d], Laura Facci[d], Emma Marcolin[d], Elena Roggiolani[a], Luca Piemontese[c], Anna Minarini[a], Morena Zusso[d],[e], Lisa Dalla Via[d], Michela Rosini[a]

[a] Department of Pharmacy and Biotechnology, Alma Mater Studiorum-University of Bologna, Via Belmeloro 6, 40126 - Bologna, Italy;

[b] Department of Molecular Medicine, University of Padova, Via G. Colombo 3, 35131 - Padova, Italy;

[c] Department of Pharmacy-Drug Sciences, University of Bari "Aldo Moro", Via E. Orabona 4, 70125 - Bari, Italy;

[d] Department of Pharmaceutical and Pharmacological Sciences, University of Padova, Via F. Marzolo 5, 35131 - Padova, Italy;

[e] IRCCS San Camillo Hospital, Via Alberoni 70, 30126 – Venezia, Italy;

Corresponding author

* Filippo Basagni: filippo.basagni2@unibo.it, Phone: +39 051 2099744.

Table of contents:

Supplementary Figures and Tables — page S2

NMR and HRMS spectra and UHPLC traces of final compounds **1**-**6** — page S6

Supplementary Figures and Tables

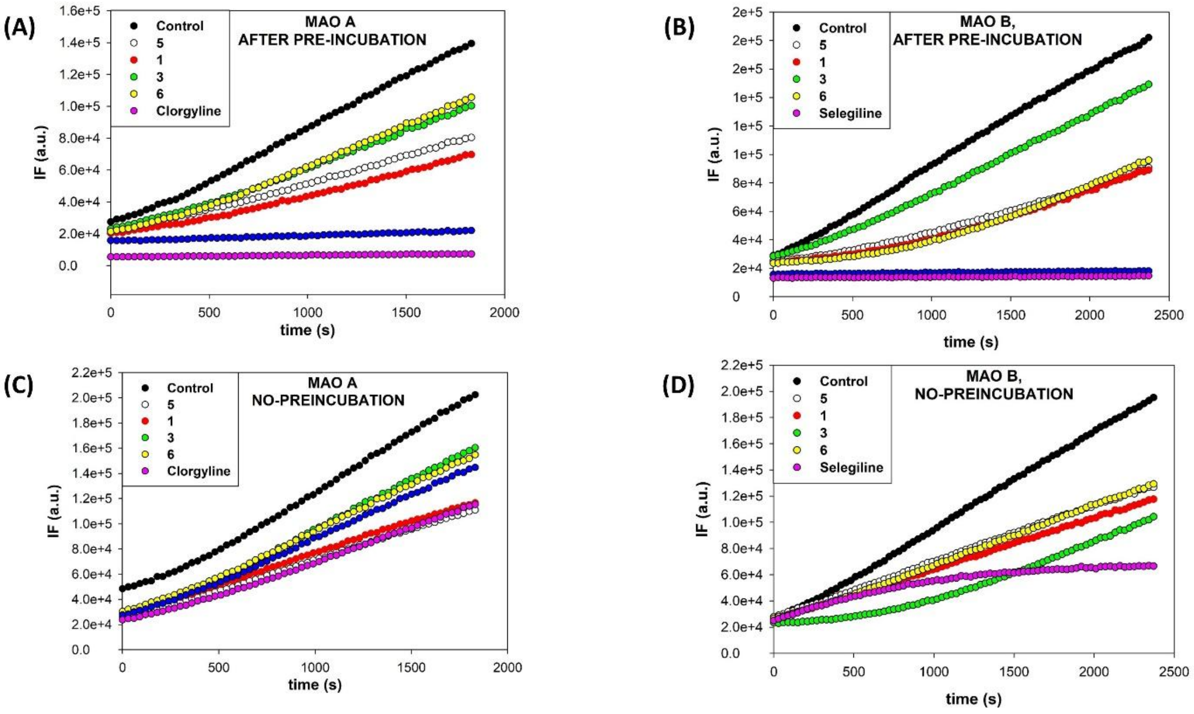

Figure S1. Representative experimental traces: recovery of enzyme activity of the pre-incubated MAO-compound solutions determined after dilution and addition of substrate (panel A and B for MAO-A and MAO-B respectively) and MAO activity of the not pre-incubated enzyme (panel C and D for MAO-A and MAO-B, respectively). The residual MAO activity was calculated in the range of maxima slope of the plot IF (a.u.) *versus* time (after a possible lag time). Substrates were: Benzylamine 10 mM for MAO-B and Tyramine 1 mM for MAO-A.

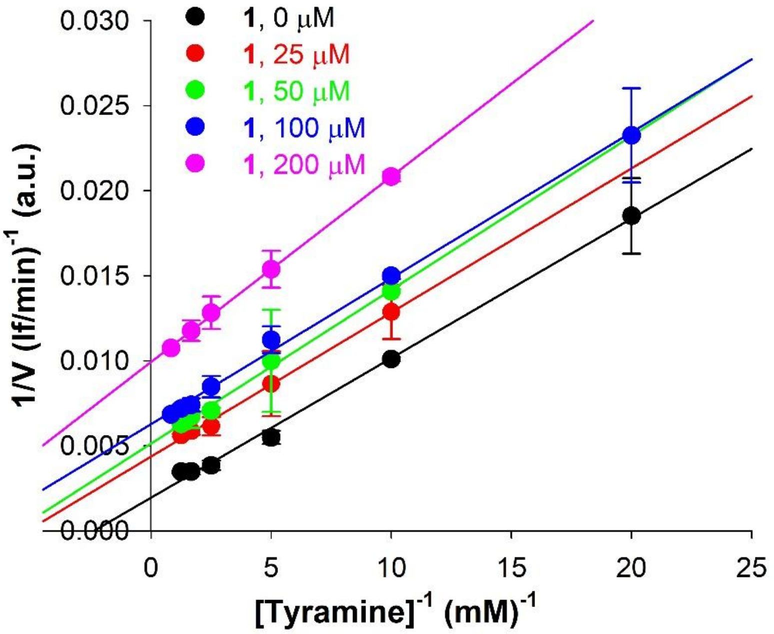

Figure S2. Mechanism of inhibition of *1* for *h*MAO-A.
Lineweaver-Burk plots of MAO-A activity in the absence (●) and in the presence of different concentrations of compound **1**. Continuous lines are the result of linear regression analysis of plotted data ($r^2 > 0.98$). The type of inhibition (uncompetitive inhibition) and the relative inhibition constant value (K_i=56±3 μM) were calculated by global fit analysis (GraphPad Prism 9.0 software).

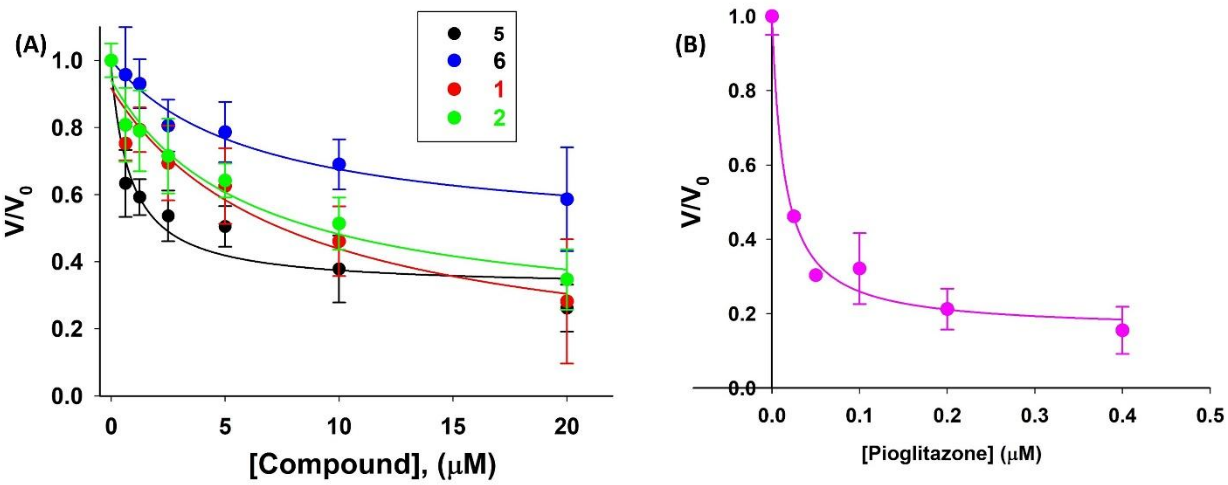

Figure S3. Effect of most active pioglitazone derivatives (A) and pioglitazone (B) on MAO activity in lysates from rat astrocytes.

The relative MAO activity (respect to control sample, v/v_0) is plotted *versus* the compound concentration to determine the IC_{50} values of the pioglitazone derivatives for the MAO expressed in rat astrocytes, which is mainly due to the MAO-B isoform.[39] Data points indicate the mean values ± SD (standard deviation) from at least three independent experiments. Continuous lines are the results of the best fitting of the "inhibitor concentration *vs* response (three parameters)" equation to the experimental data, by using the GraphPad Prism 9.0 software. The use of the third parameter in the fitting equation was necessary to take into account the contribution of the MAO-A isoform to the total MAO activity, which was found to be not affected by these compounds as for the human isoform. Indeed, the asymptotic residual activity found at high concentration of all compounds (about 30% for **1**, **2** and **5**; 45% for **6** and 10% for Pioglitazone) represents the MAO activity that cannot be inhibited by the compound. The IC_{50} values calculated are reported in Table 2.

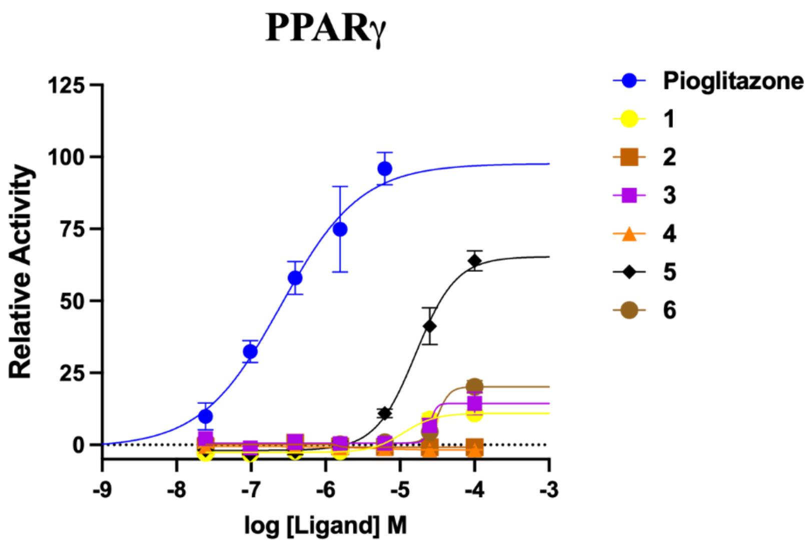

Figure S4. PPARγ transactivation assay. Representative dose-response curves obtained by luciferase reporter assay in transiently transfected HepG2 cells for compounds 1–6, and pioglitazone (reference agonist).

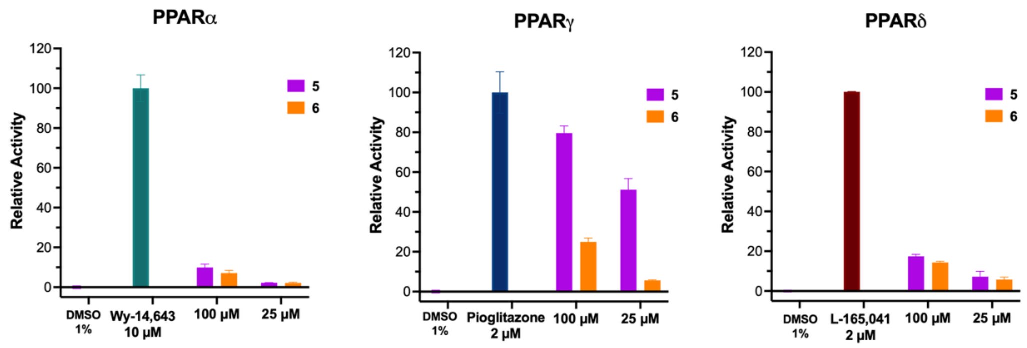

Figure S5. Biological activity of compounds 5 and 6 on PPARα, PPARγ and PPARδ with WY-14,643, pioglitazone and L-165,041 as reference agonists.

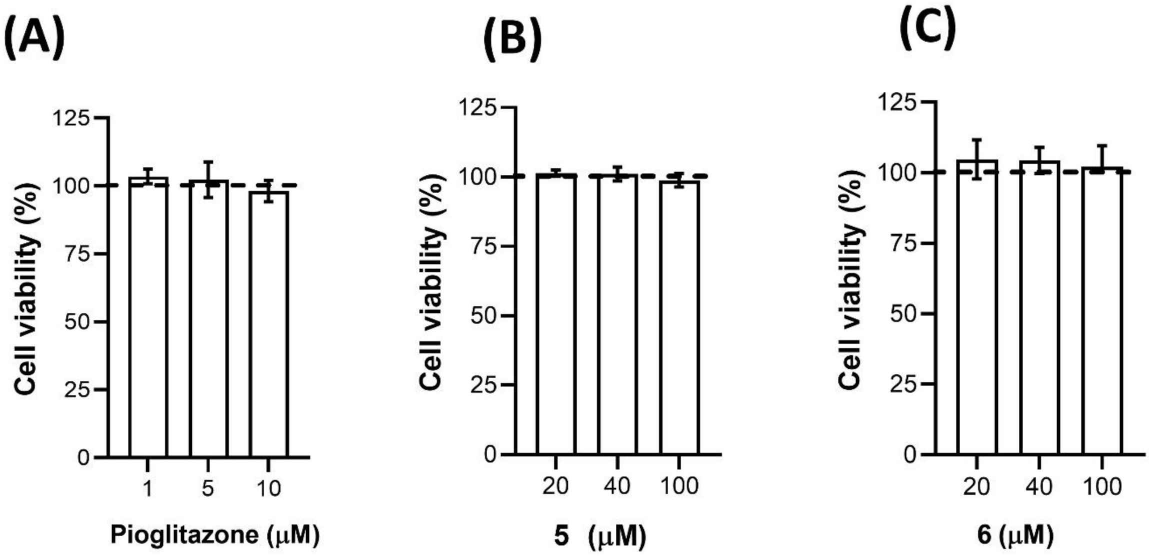

Figure S6. Viability of astrocyte-enriched cultures after treatment with Pioglitazone (A), 5 (B) and 6 (C).
Cells were cultured in medium containing 10% of serum, which was replaced with serum-free medium before 24h treatment with increasing concentrations of pioglitazone (1-10 μM) (**A**), **5** (**B**), and **6** (**C**) (20-100 μM). At the end of incubation period cell viability was determined using the sulforhodamine B (SRB) assay. Results are expressed as percentage of cell viability relative to control cells (dashed lines). Data are means ± SEM of three independent experiments.

Table S1. Cell viability of selected compounds at 20 μM after 48h of treatment in two different types of human cells.
Values are expressed as mean ± SD of data from at least three experiments in duplicate.

**Compound (20μM)**	**Cell Viability (%)**	
	**LN229**	**C20**
**1**	97 ± 3	95 ± 4
**2**	97 ± 5	90 ± 3
**5**	96 ± 7	98 ± 5
**6**	89 ± 2	98 ± 4
**Pioglitazone**	100 ± 2	98 ± 5

Table S2. Effect of selected compounds on the DCF oxidation rate induced by ABIP.
Values are expressed as mean ± SD

**Compound[a]**	**DCF oxidation rate relative to control sample (%)**
**Pioglitazone**	100 ± 2
**1**	47 ± 5
**2**	102 ± 5
**5**	65 ± 10
**6**	101 ± 2
**Quercetin**	1 ± 1

[a] Compounds were used at 5 μM and quercetin at 1 μM.

NMR and HRMS spectra and UHPLC traces of final compounds 1-6

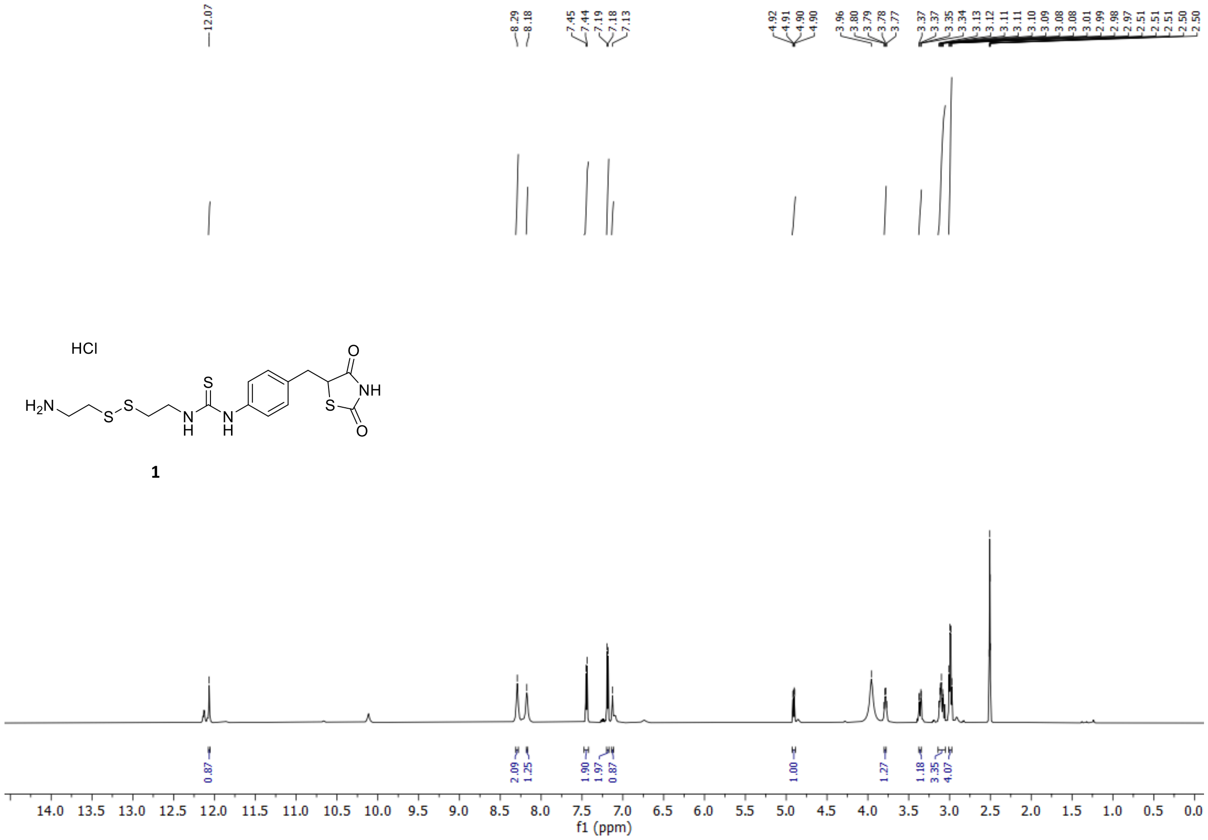

1

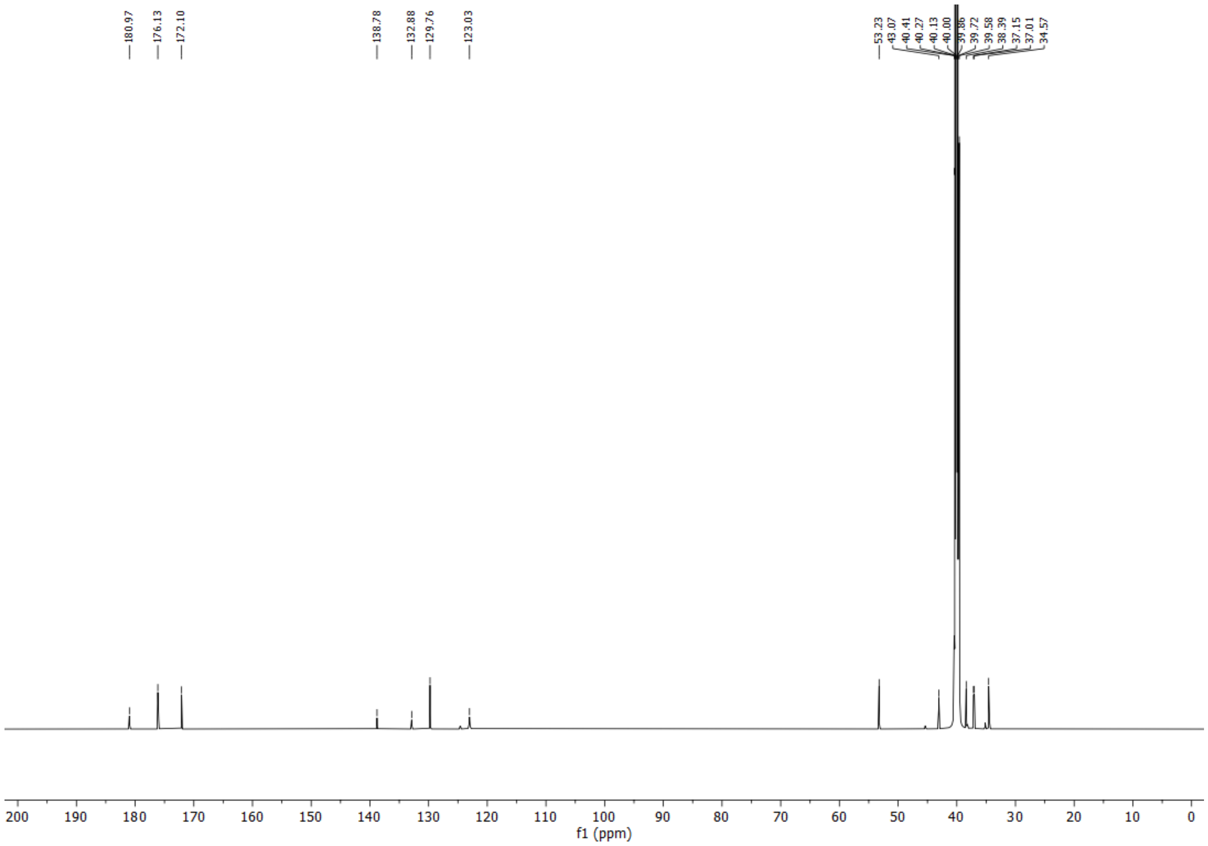

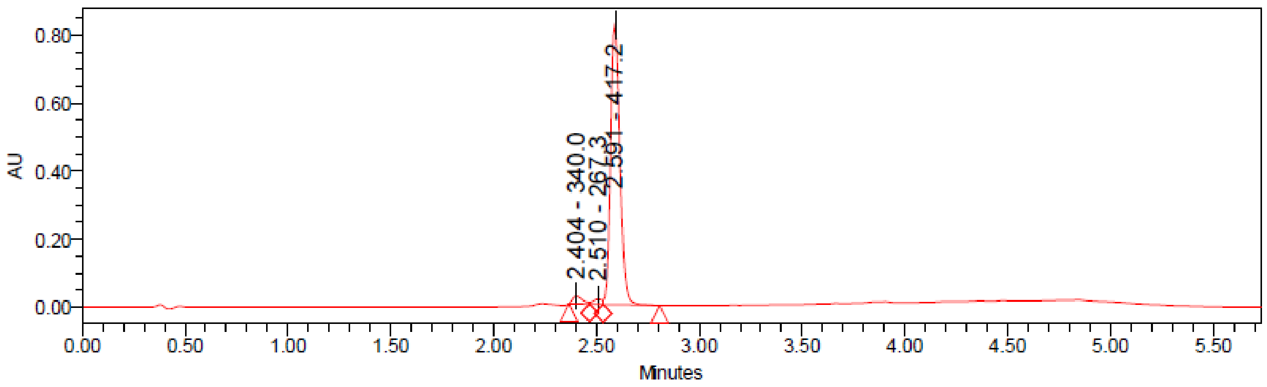

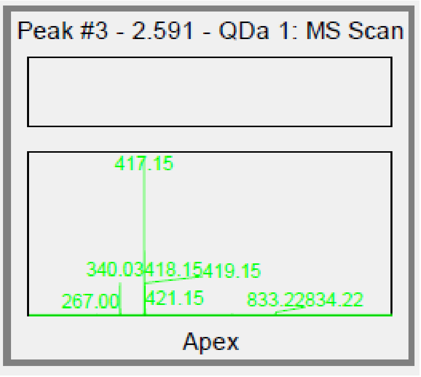

Peak Results

	Name	RT	Area	% Area	Height
1		2.404	80625	2.97	23915
2		2.510	48170	1.77	16313
3		2.591	2587832	95.26	823769

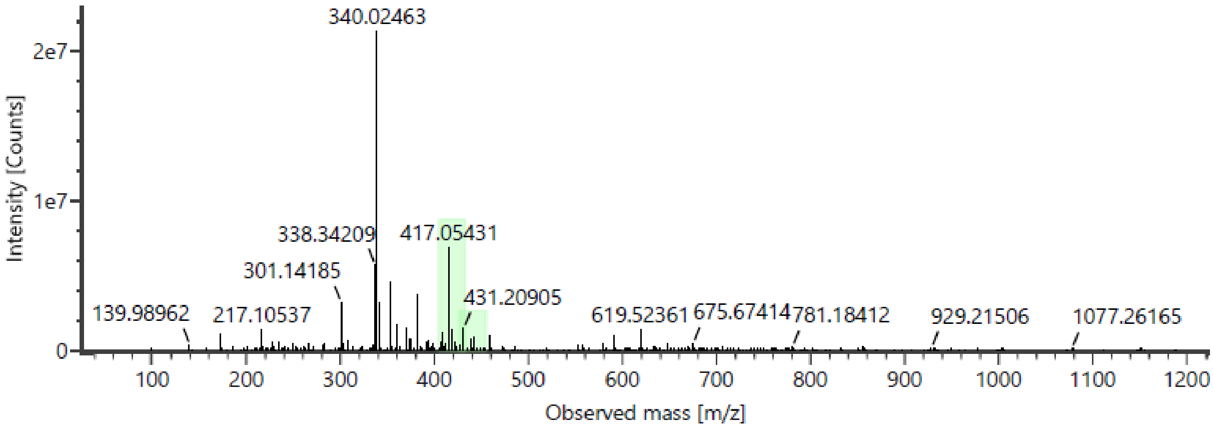

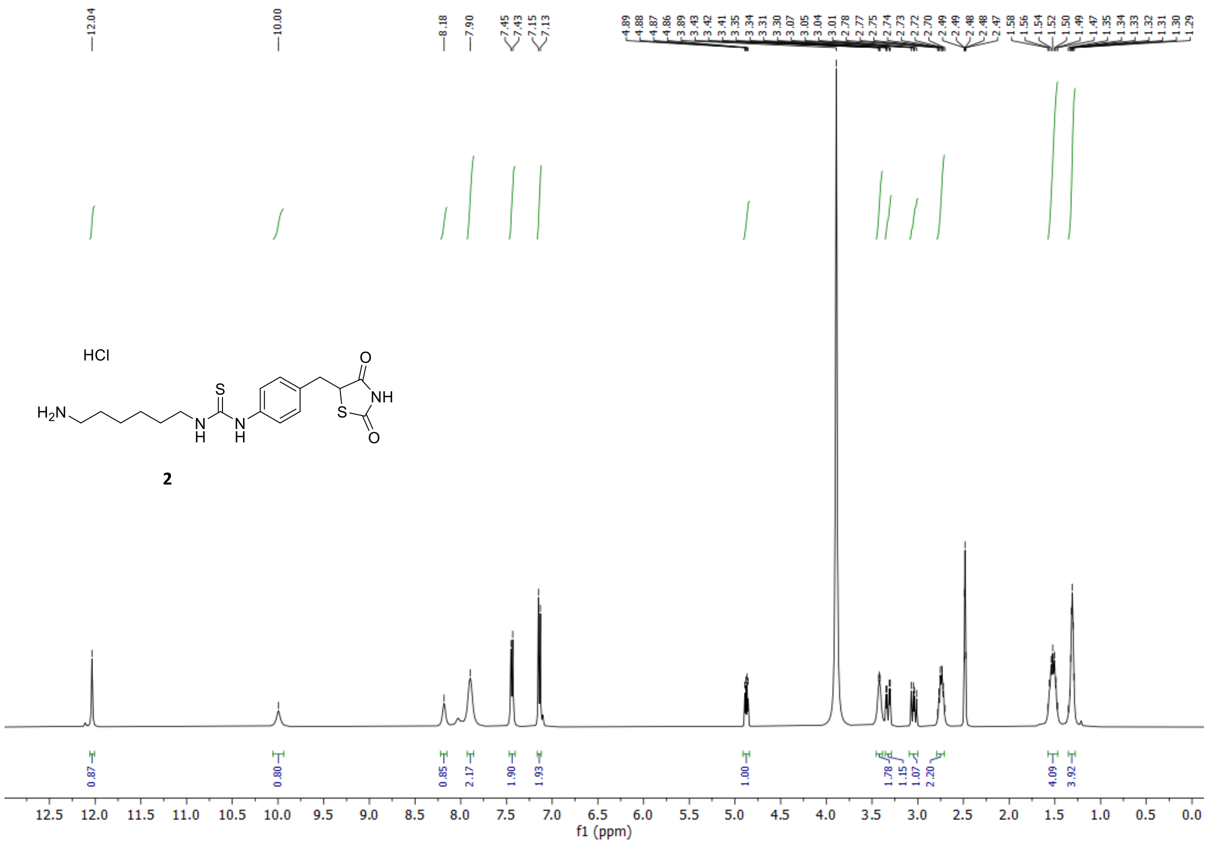

12.04
10.00
8.18
7.90
7.45
7.43
7.15
7.13
4.89
4.88
4.87
4.86
3.89
3.43
3.42
3.41
3.35
3.34
3.31
3.30
3.07
3.05
3.04
3.01
2.78
2.77
2.75
2.74
2.73
2.72
2.70
2.49
2.49
2.48
2.48
2.47
1.58
1.56
1.54
1.52
1.50
1.49
1.47
1.35
1.34
1.33
1.32
1.31
1.30
1.29
HCl
H_2N
S
N
H
NH
O
2
0.87
0.80
0.85
2.17
1.90
1.93
1.00
1.78
1.15
1.07
2.20
4.09
3.92
f1 (ppm)

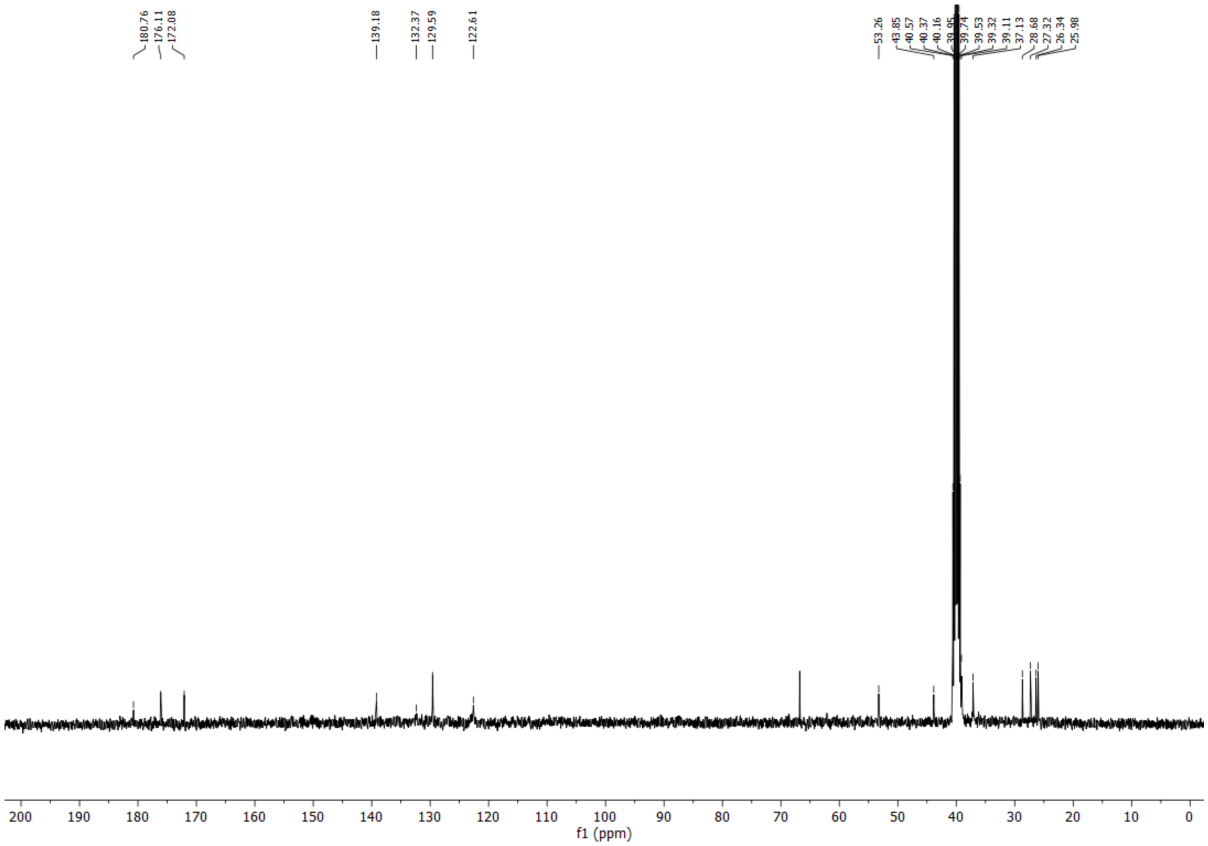

180.76
176.11
172.08
139.18
132.37
129.59
122.61
53.26
43.85
40.57
40.37
40.16
39.74
39.53
39.32
39.11
37.13
28.68
27.32
26.34
25.98
f1 (ppm)

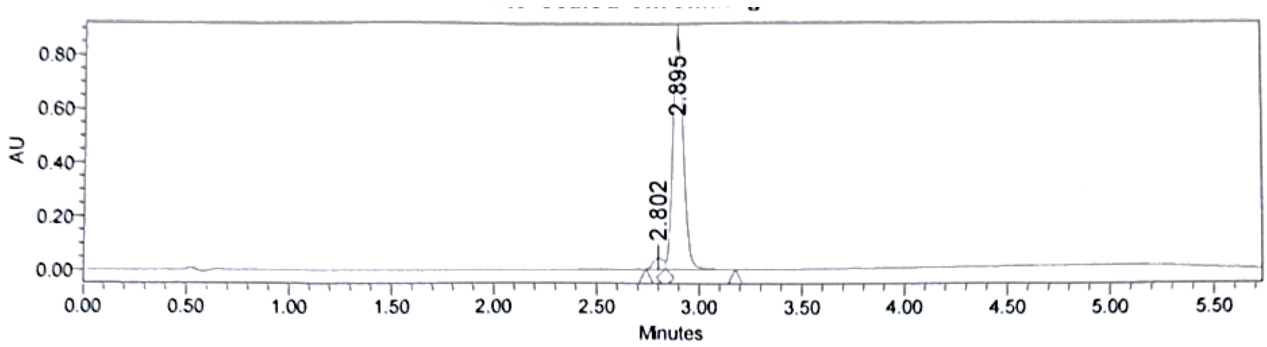

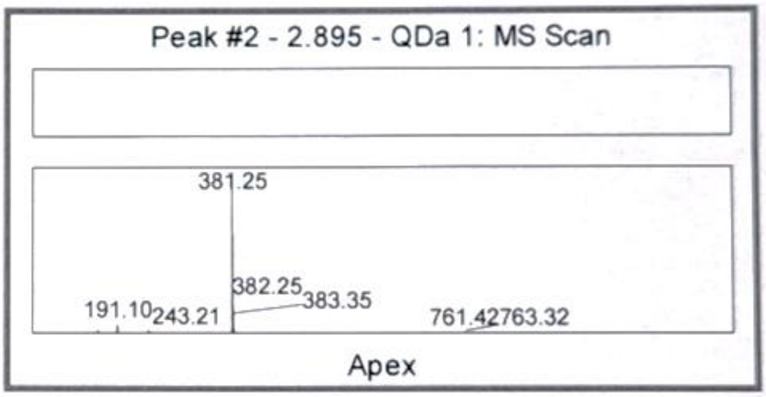

Peak Results

	Name	RT	Area	Height	% Area
1		2.802	145939	42653	4.56
2		2.895	3055862	875693	95.44

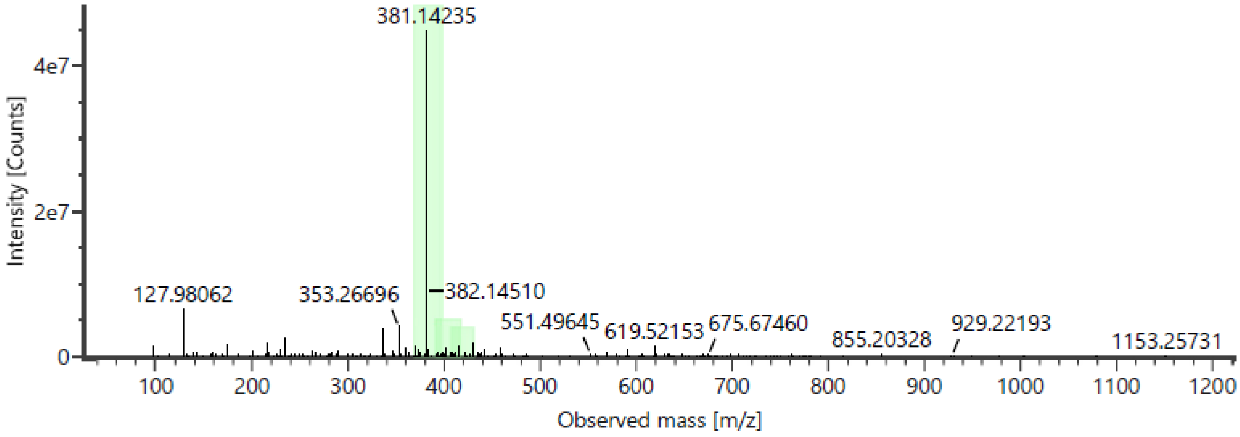

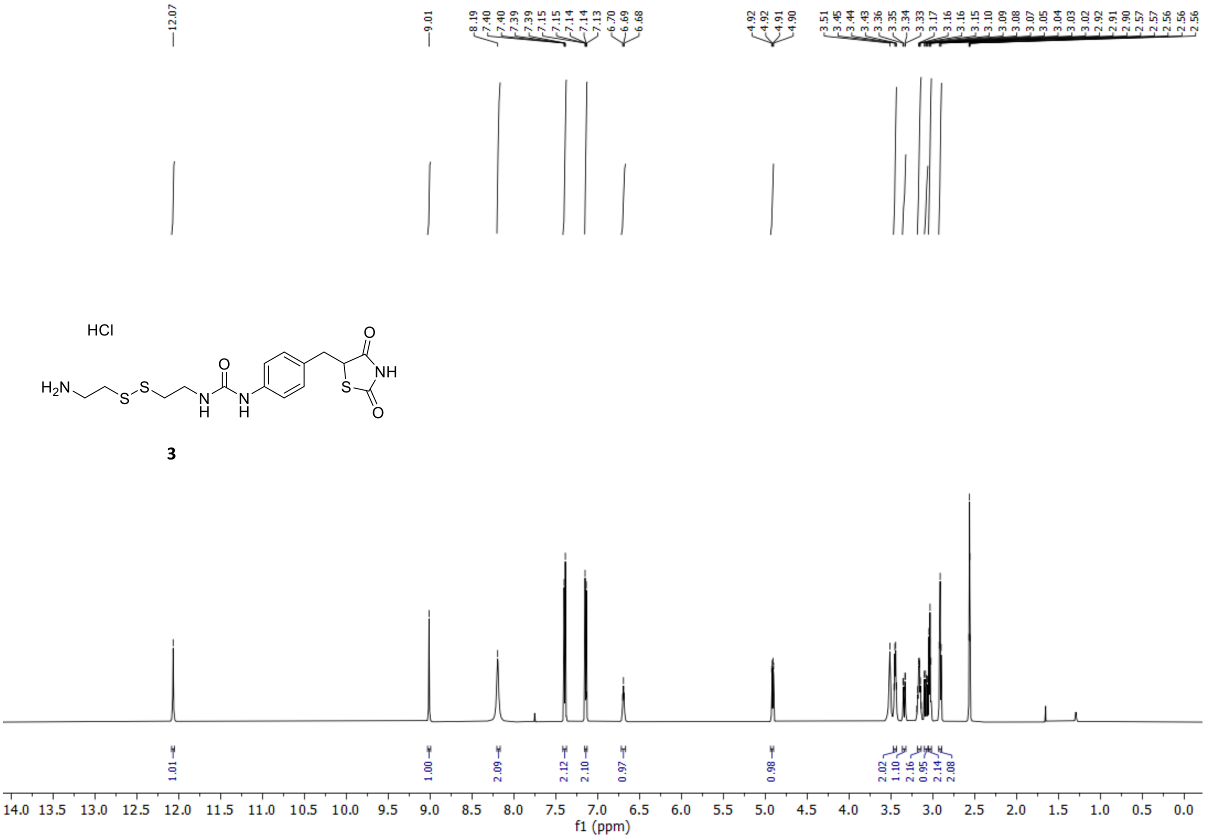
12.07
9.01
8.19 7.40 7.40 7.39 7.39 7.15 7.15 7.14 7.13 6.70 6.69 6.68
4.92 4.92 4.91 4.90
3.51 3.45 3.44 3.43 3.36 3.35 3.34 3.33 3.17 3.16 3.16 3.15 3.10 3.09 3.08 3.07 3.05 3.04 3.03 3.02 2.92 2.91 2.90 2.57 2.57 2.56 2.56
1.01 1.00 2.09 2.12 2.10 0.97 0.98 2.02 1.10 2.16 0.95 2.14 2.08
14.0 13.5 13.0 12.5 12.0 11.5 11.0 10.5 10.0 9.5 9.0 8.5 8.0 7.5 7.0 6.5 6.0 5.5 5.0 4.5 4.0 3.5 3.0 2.5 2.0 1.5 1.0 0.5 0.0
f1 (ppm)
HCl
H2N
S
S
N H
O
N H
S
NH
O
O

3

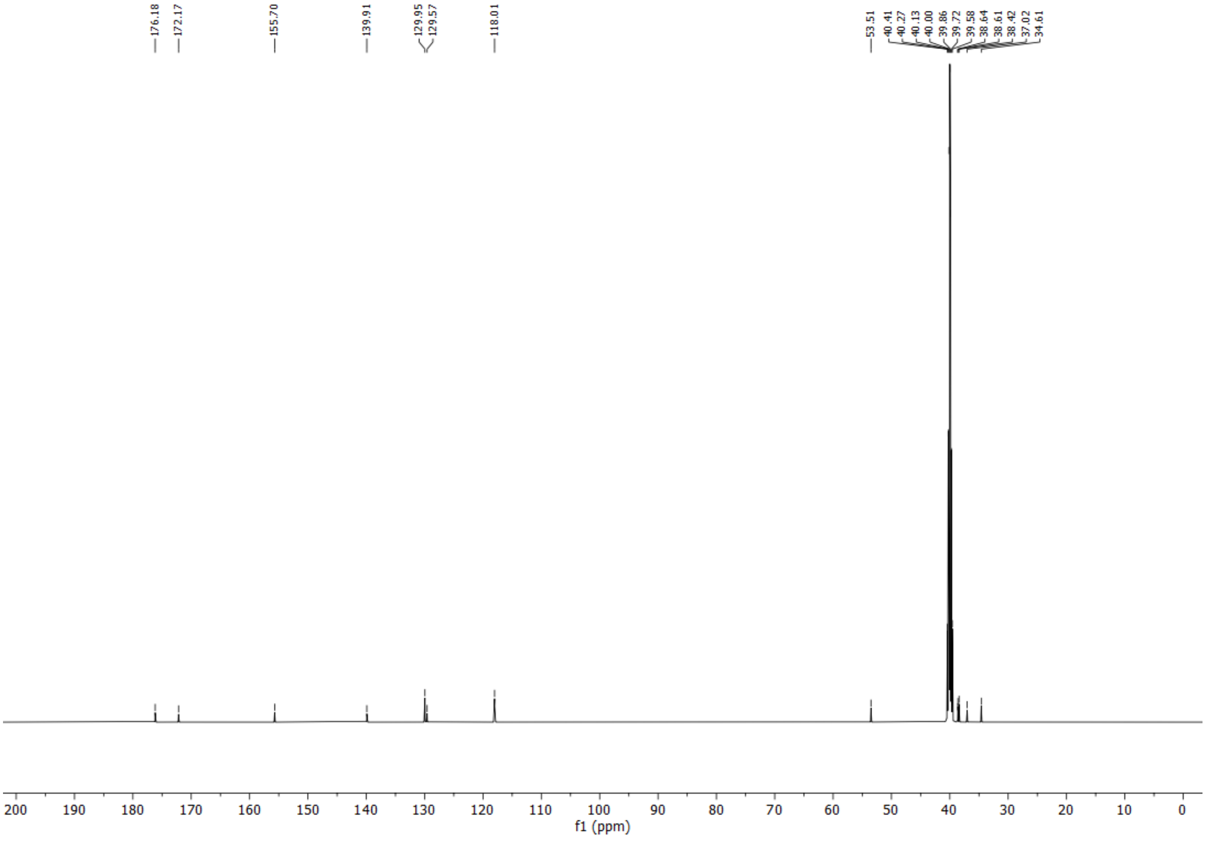
176.18 172.17 155.70 139.91 129.95 129.57 118.01
53.51 40.41 40.27 40.13 40.00 39.86 39.72 39.58 38.64 38.61 38.42 37.02 34.61
200 190 180 170 160 150 140 130 120 110 100 90 80 70 60 50 40 30 20 10 0
f1 (ppm)

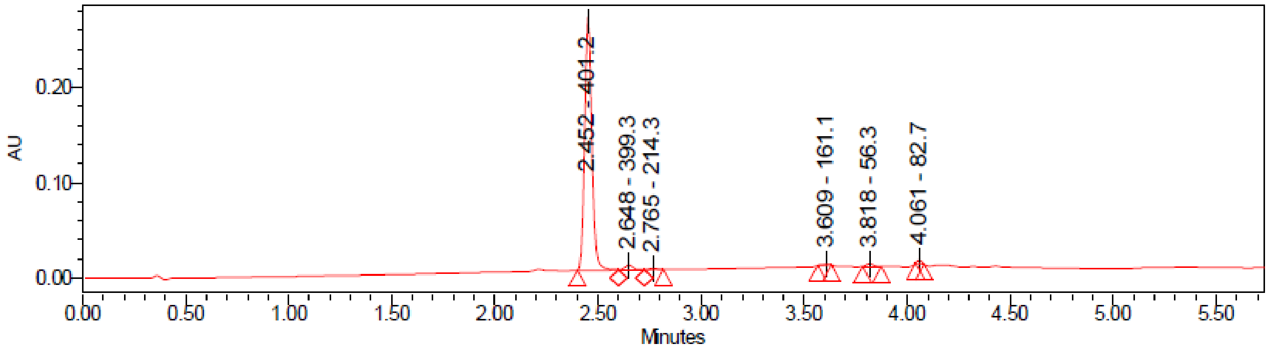

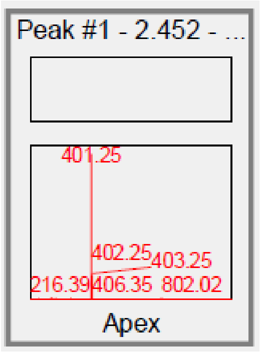

Peak Results

	Name	RT	Area	% Area	Height
1		2.452	660589	95.46	262288
2		2.648	13504	1.95	4986
3		2.765	3066	0.44	1246
4		3.609	2955	0.43	1216
5		3.818	6621	0.96	2827
6		4.061	5271	0.76	3457

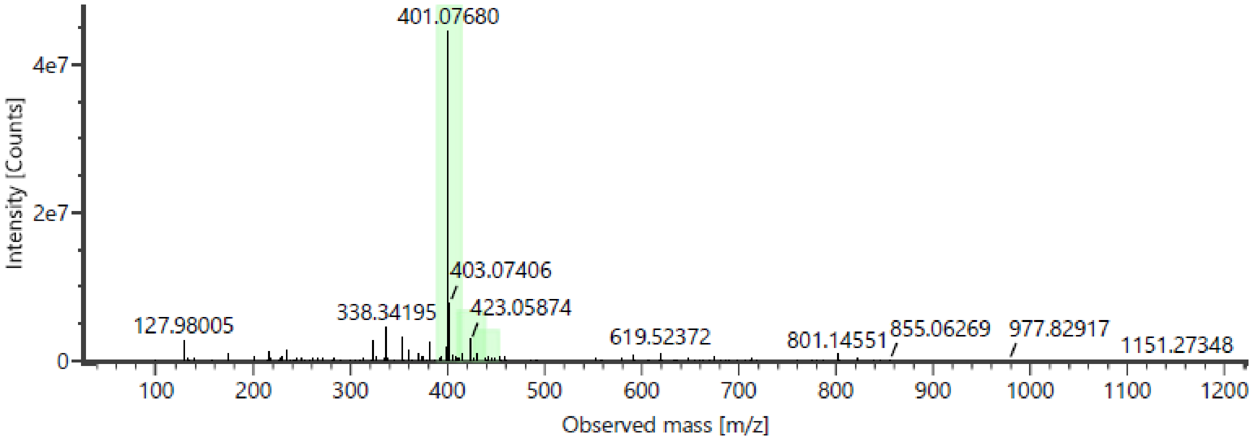

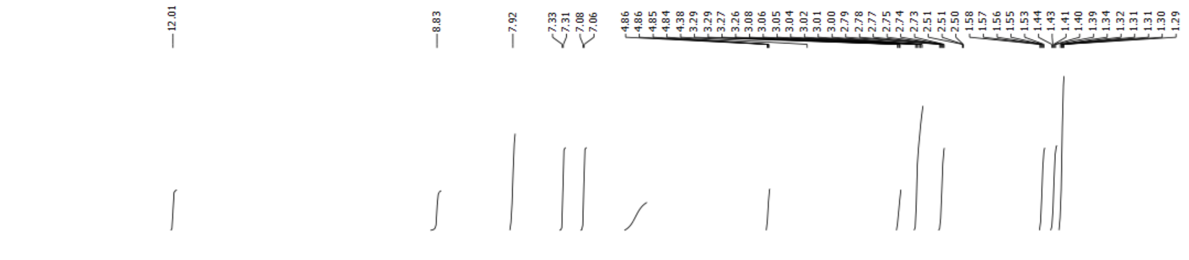

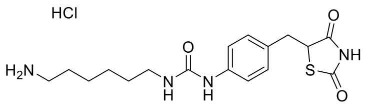
HCl
H2N
O
N
H
N
H
S
NH
O
O

4

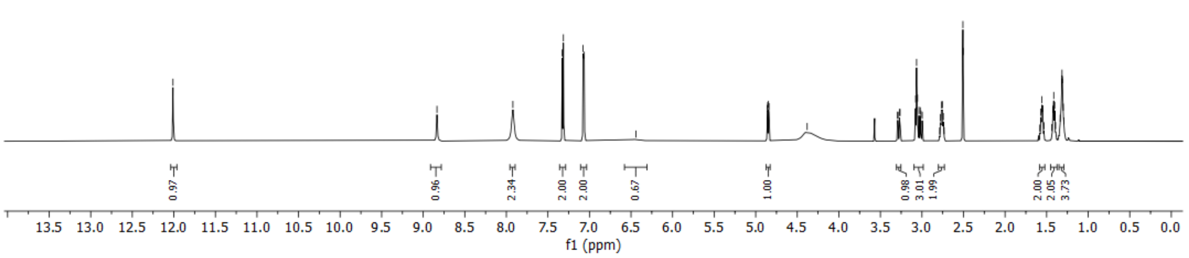
12.01
8.83
7.92
7.33
7.31
7.08
7.06
4.86
4.86
4.85
4.84
4.38
3.29
3.29
3.27
3.26
3.08
3.06
3.05
3.04
3.02
3.01
3.00
2.79
2.78
2.77
2.75
2.74
2.73
2.51
2.51
2.50
1.58
1.57
1.56
1.55
1.53
1.44
1.43
1.41
1.40
1.39
1.34
1.32
1.31
1.31
1.30
1.29
0.97
0.96
2.34
2.00
2.00
0.67
1.00
0.98
3.01
1.99
2.00
2.05
3.73
f1 (ppm)

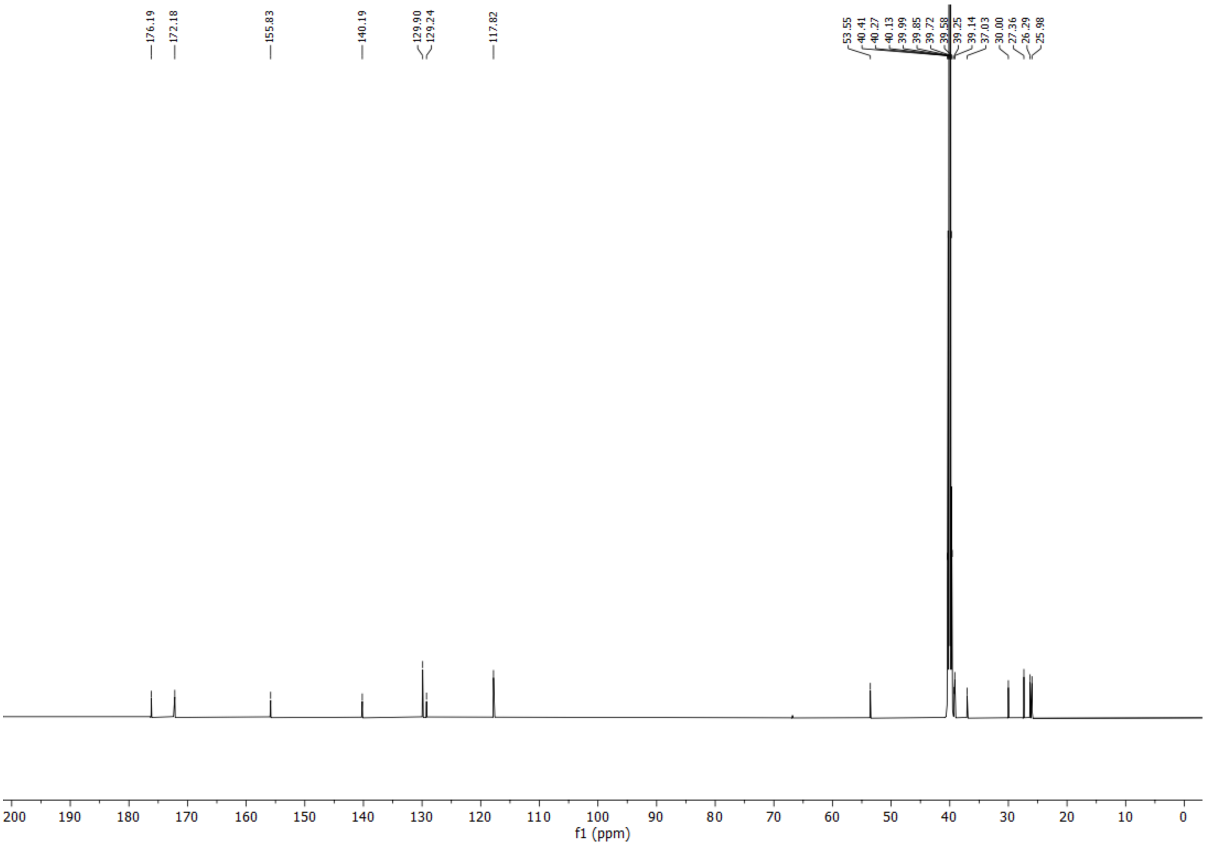
176.19
172.18
155.83
140.19
129.90
129.24
117.82
53.55
40.41
40.27
40.13
39.99
39.85
39.72
39.58
39.25
39.14
37.03
30.00
27.36
26.29
25.98
f1 (ppm)

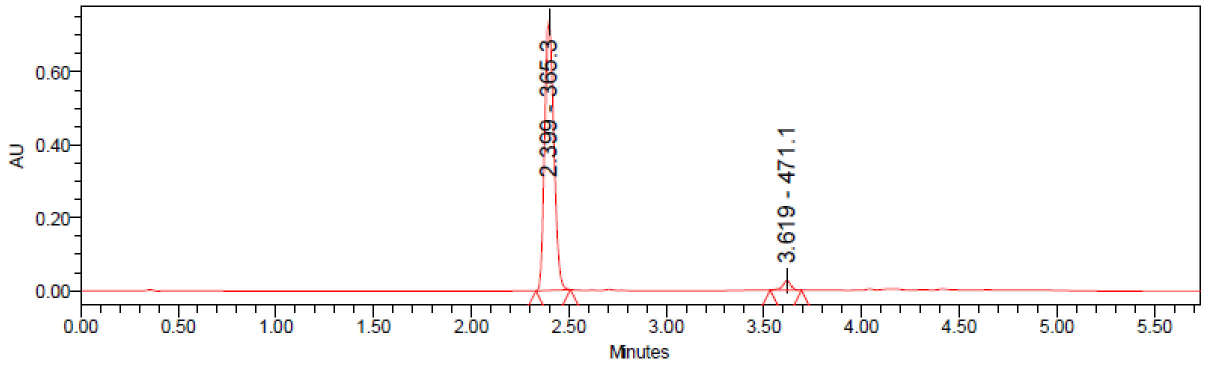

Peak Results

	Name	RT	Area	% Area	Height
1		2.399	2349628	96.86	737482
2		3.619	76123	3.14	26682

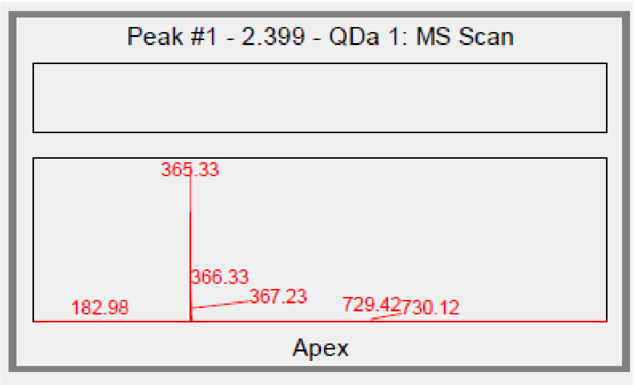

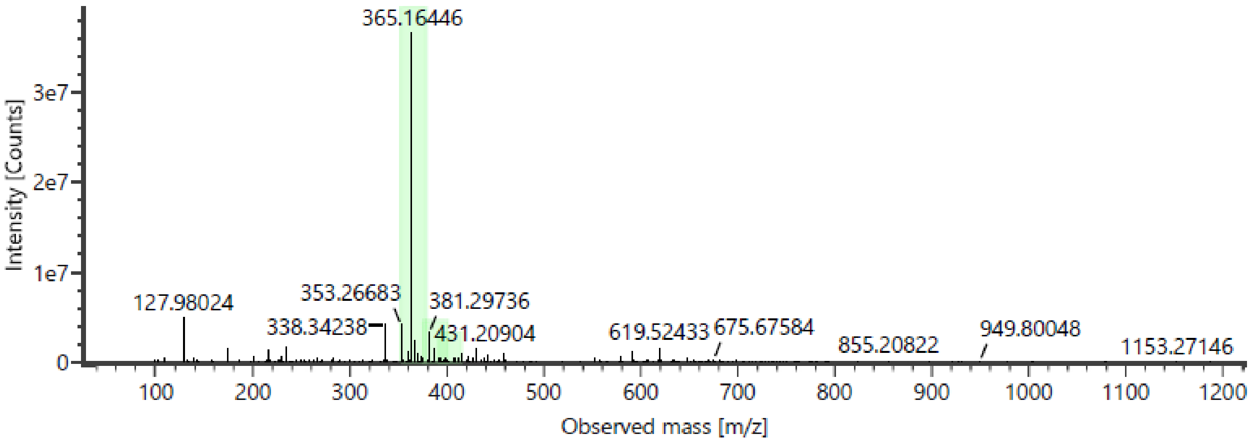

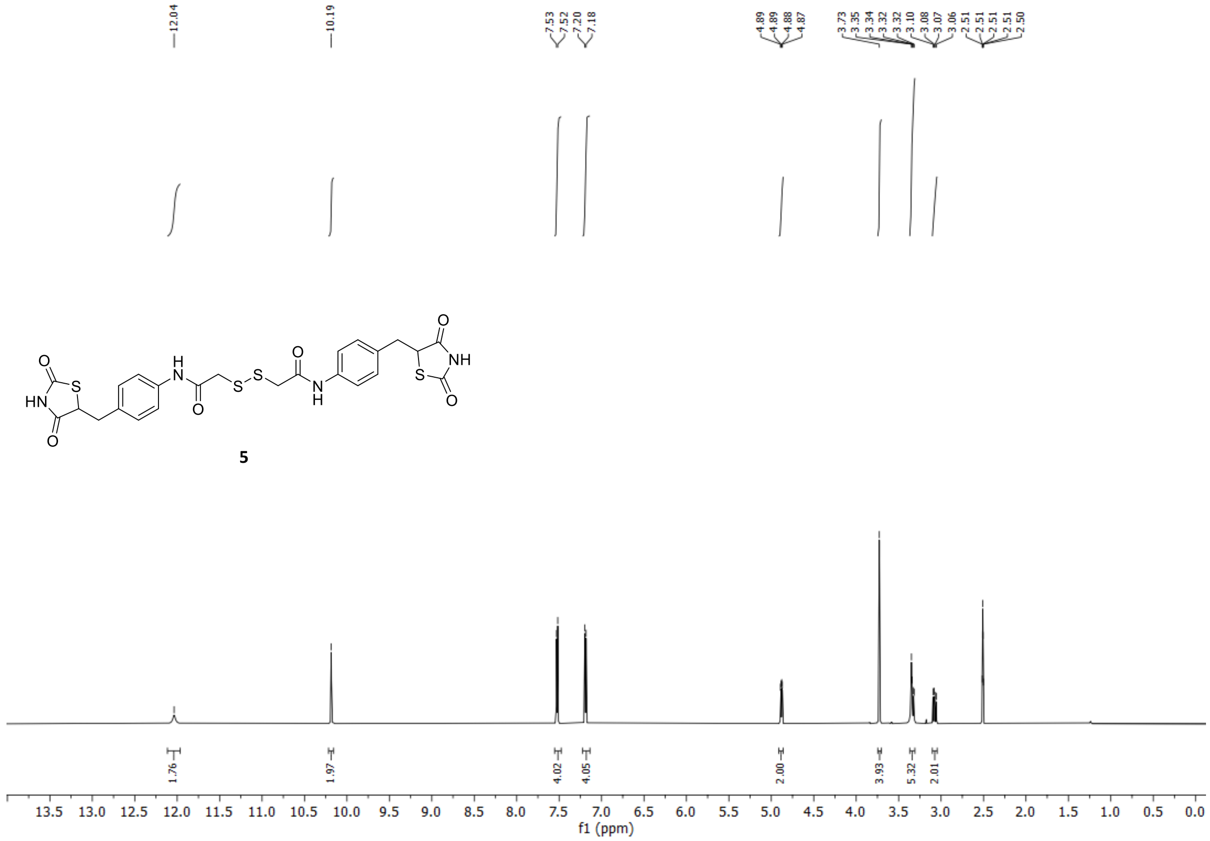

12.04
10.19
7.53
7.52
7.20
7.18
4.89
4.89
4.88
4.87
3.73
3.35
3.34
3.32
3.32
3.10
3.08
3.07
3.06
2.51
2.51
2.51
2.51
2.50
O
HN
S
O
H
N
O
S
S
O
N
H
O
NH
S
O
5
1.76
1.97
4.02
4.05
2.00
3.93
5.32
2.01
13.5 13.0 12.5 12.0 11.5 11.0 10.5 10.0 9.5 9.0 8.5 8.0 7.5 7.0 6.5 6.0 5.5 5.0 4.5 4.0 3.5 3.0 2.5 2.0 1.5 1.0 0.5 0.0
f1 (ppm)

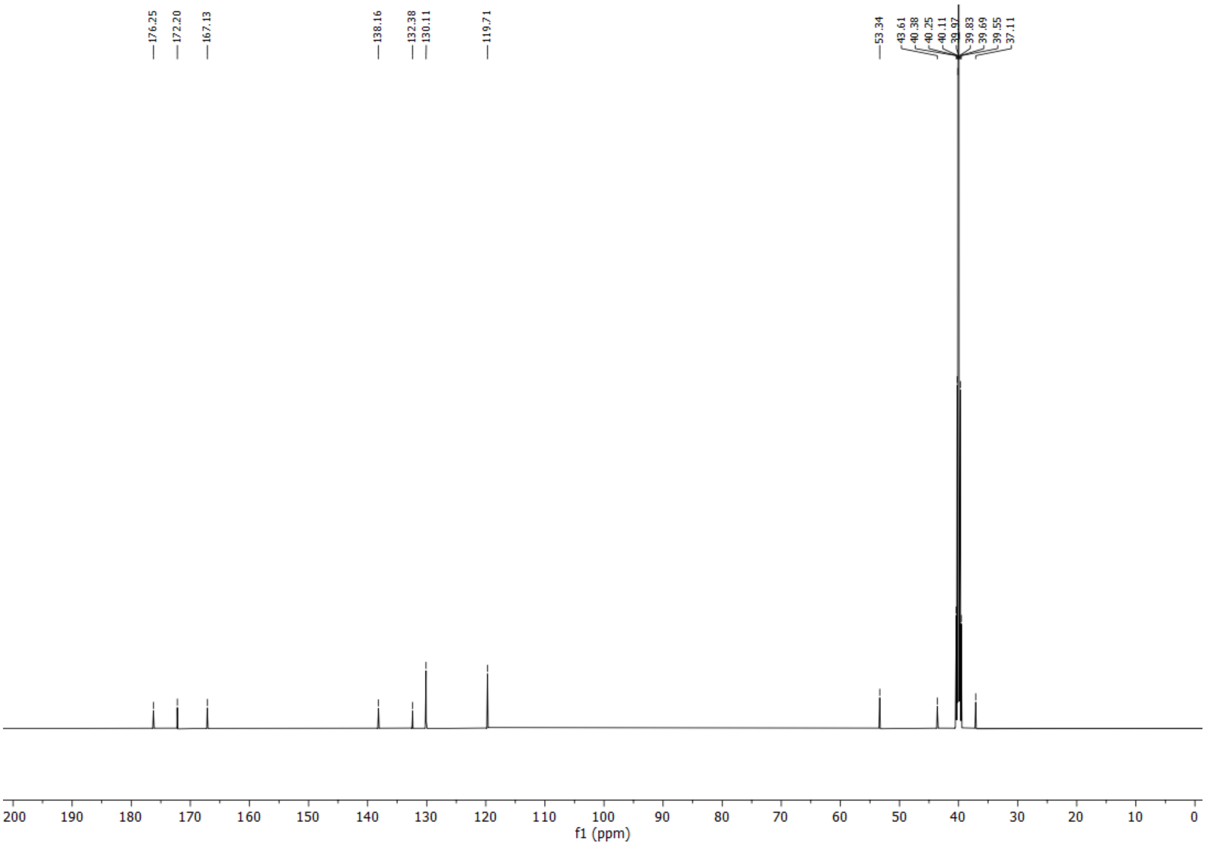

176.25
172.20
167.13
138.16
132.38
130.11
119.71
53.34
43.61
40.38
40.25
40.11
39.97
39.83
39.69
39.55
37.11
200 190 180 170 160 150 140 130 120 110 100 90 80 70 60 50 40 30 20 10 0
f1 (ppm)

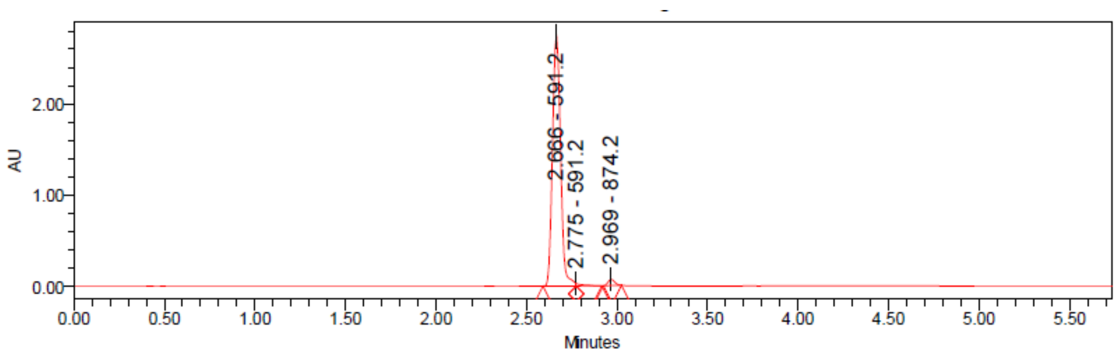

Peak Results

	Name	RT	Area	% Area	Height
1		2.666	8511540	97.27	2742261
2		2.775	68339	0.78	27607
3		2.969	170584	1.95	67262

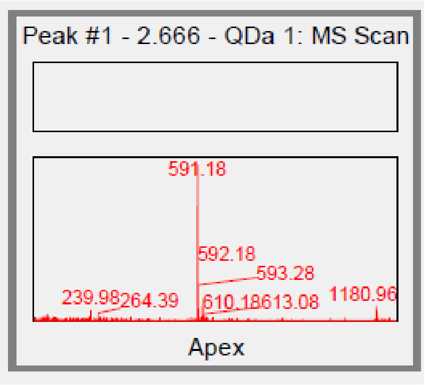

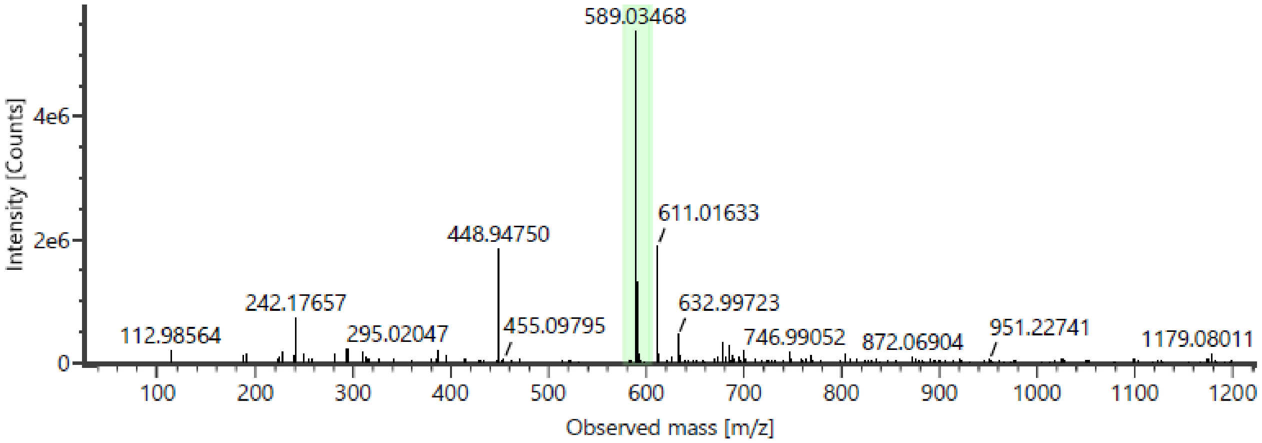

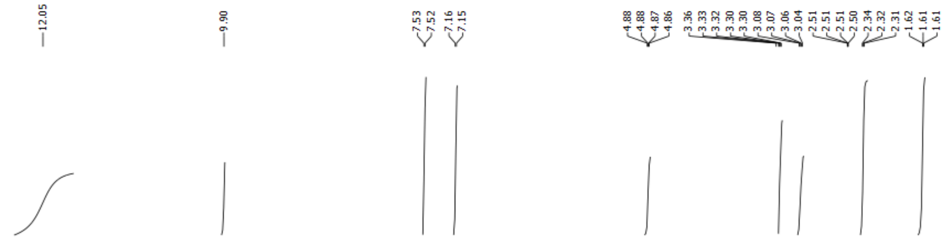

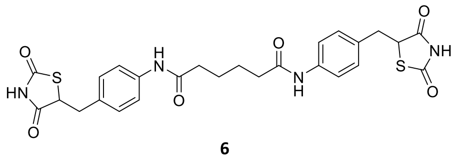

6

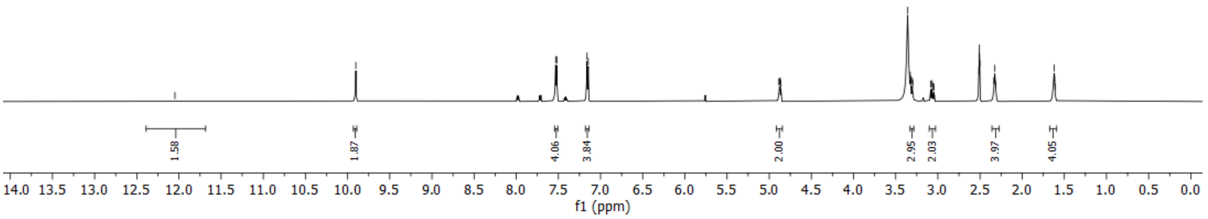

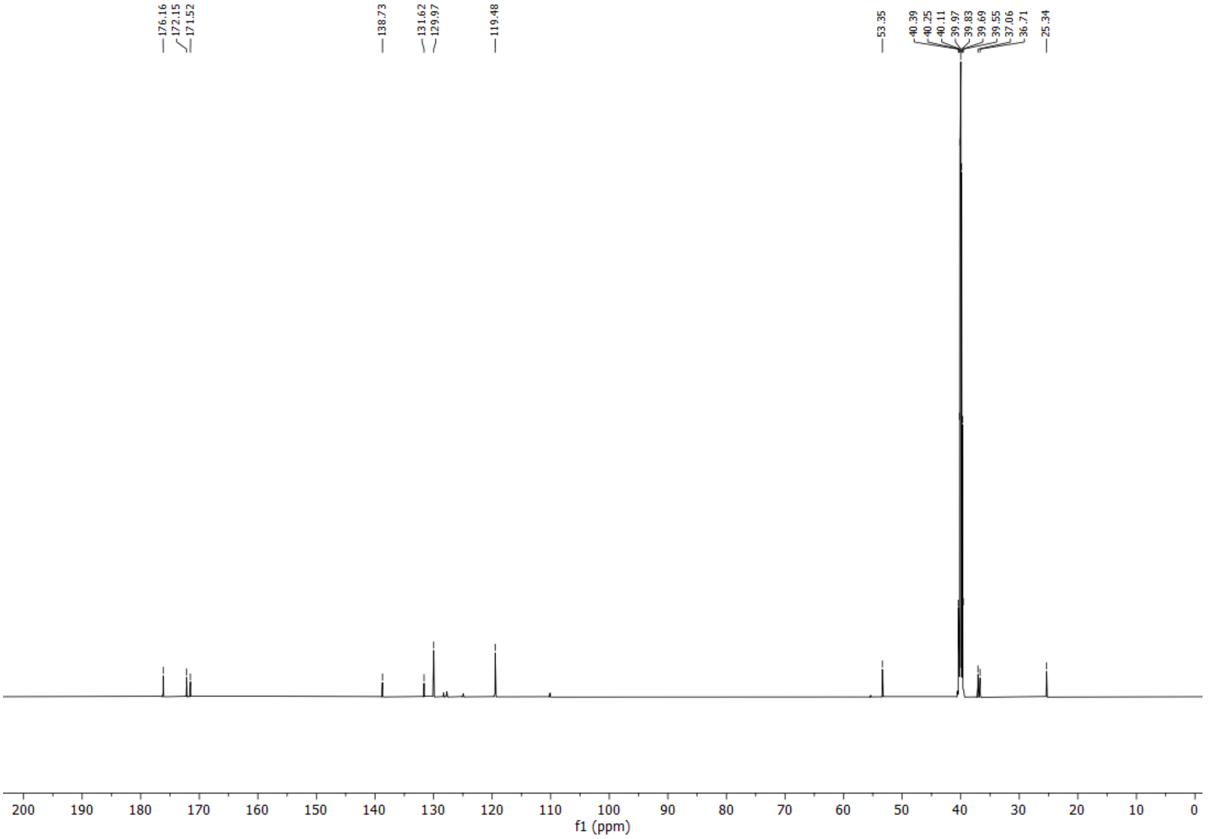

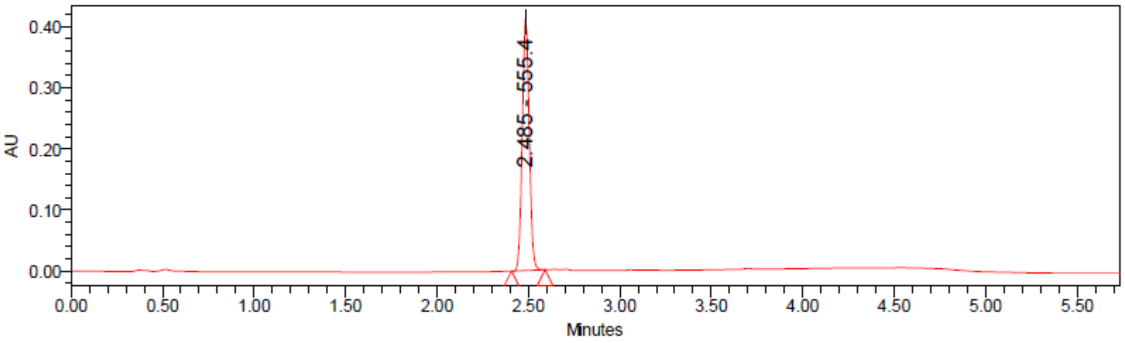

Peak Results

	Name	RT	Area	% Area	Height
1		2.485	1099157	100.00	408868

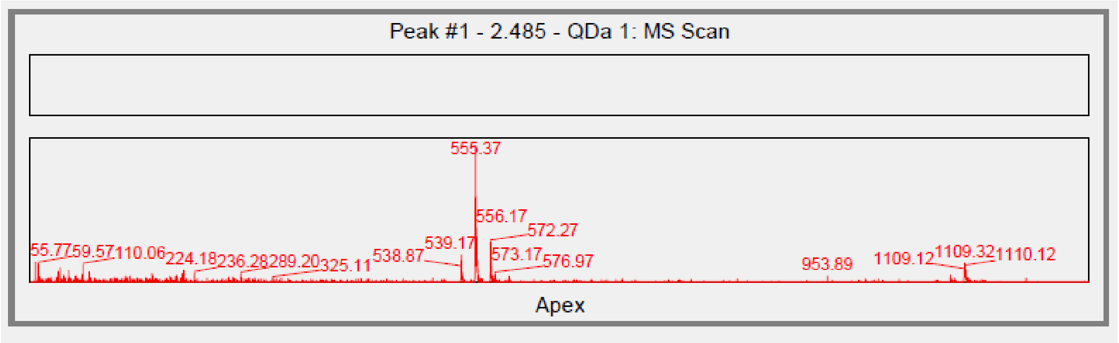

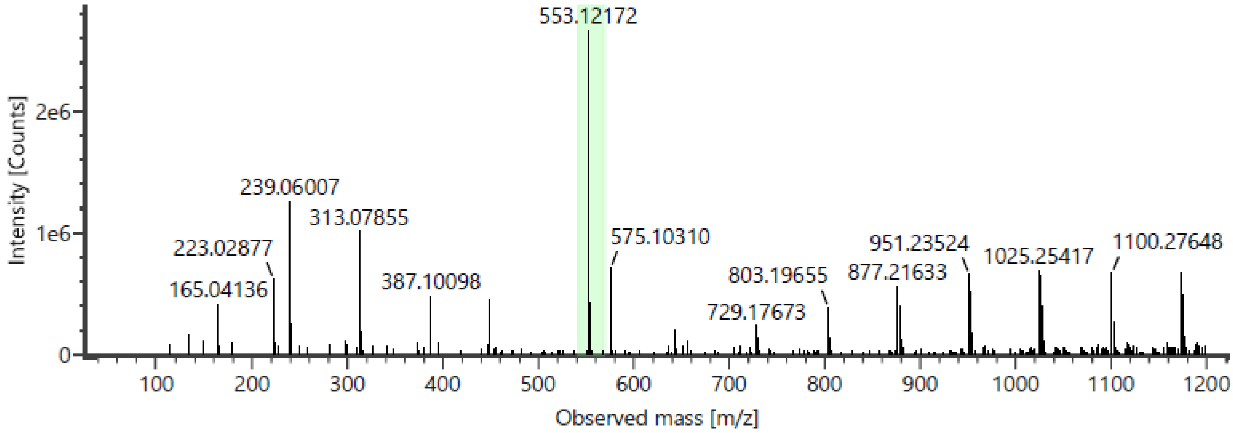

Reference

[39] Levitt, P., Pintar, J. E., Breakefield, X. O., "Immunocytochemical demonstration of monoamine oxidase B in brain astrocytes and serotonergic neurons," *Proc Natl Acad Sci U S A* 79, no. 20 (1982), https://doi.org/10.1073/pnas.79.20.6385.
